# Plasmonic Su–Schrieffer–Heeger chains with strong coupling amplitudes

**DOI:** 10.1126/sciadv.aea3844

**Published:** 2025-12-10

**Authors:** Benedikt Schurr, Matthias Hensen, Luisa Brenneis, Philipp Kessler, Jin Qin, Victor Lisinetskii, Ronny Thomale, Tobias Brixner, Bert Hecht

**Affiliations:** ^1^ NanoOptics & Biophotonics Group, Experimental Physics 5, University of Würzburg, Am Hubland, 97074 Würzburg, Germany.; ^2^ Institut für Physikalische und Theoretische Chemie, Universität Würzburg, Am Hubland, 97074 Würzburg, Germany.; ^3^ Institute for Theoretical Physics and Astrophysics, University of Würzburg, 97074 Würzburg, Germany.

## Abstract

Plasmonic many-particle systems with precisely tuned resonances and coupling strengths can exhibit emergent collective properties governed by universal principles. In one-dimensional chains with alternating couplings, known as Su–Schrieffer–Heeger (SSH) systems, this includes the formation of topologically protected mid-gap modes whose intensities localize at the chain’s ends. This subwavelength localization at optical frequencies is crucial for achieving strong coupling of mid-gap modes to two-level systems under ambient conditions, extending topological protection to hybrid light–matter states. Here, we have fabricated SSH chains from plasmonic nanoslit resonators with strong interresonator coupling. The alternating distance between the nanoslit resonators is controlled with subnanometer precision, enabling accurate prediction and experimental observation of topologically protected mid-gap modes via photoemission electron microscopy. Our results open the path toward experimental realizations of two-dimensional photonic metasurfaces exhibiting higher-order topological modes that can be strongly coupled to single emitters and quantum materials at ambient conditions.

## Teaser

Strong coupling enables device-relevant spectral separation of broad, topologically protected mid-gap modes from band edges.

## INTRODUCTION

Topological photonics has emerged as a vibrant research field, offering new paradigms for controlling light at the nanoscale by leveraging concepts from condensed matter physics (*1*). Among these, the Su–Schrieffer–Heeger (SSH) model, describing a one-dimensional (1D) topological insulator, has been widely explored for its ability to host robust edge states protected by a topological invariant (*2*). In terms of energy, these collective edge-state modes lie in a bandgap of the system and are therefore also called mid-gap modes. Originally developed to describe electron behavior in polyacetylene (*3*), the SSH model has since been extended to photonic systems, where alternating couplings between resonators or waveguides produce mid-gap modes that, although involving all particles of the chain, exhibit spatially localized intensity at system edges (*4*–*6*). These mid-gap modes are particularly appealing for applications in robust light transport and quantum information processing owing to their resilience against perturbations. Plasmonic platforms, which enable deep subwavelength confinement of light, provide an ideal testbed for exploring topological phenomena at the nanoscale and offer the intriguing possibility to achieve strong coupling to quantum emitters at ambient conditions (*7*, *8*). Furthermore, the mode localization of the mid-gap modes at the respective ends of the chain might be suited to establish a strong coupling of quantum systems by means of a topologically protected mode over distances larger than the working wavelength of the system (*9*).

Prior work was aimed at demonstrating plasmonic SSH chains and their characteristic mid-gap modes using linear disc chains (*10*). There, however, overlapping particles and widely spaced particles were used to increase the coupling strength contrast needed to energetically separate the mid-gap modes from the band-edge modes. This approach compromises the identity of individual resonators so that no collective modes, and thus no mid-gap modes, can exist at all on the basis of identical chain links. Very recently, Yan *et al.* (*11*) have demonstrated near-field imaging of topological edge-state modes in plasmonic chains of gold nanodiscs connected by waveguides of varying widths. However, in this case, strong screening effects reduce the coupling strength, leading to a narrowed bandgap and causing possible topological mid-gap modes to mix with energetically nearby trivial states. Notomi and colleagues (*12*) have used optical far-field microscopy to study plasmonic zigzag chains exploiting alternating J- and H-like couplings between induced electric dipoles in neighboring metal discs. However, an increased light scattering signal at the ends of the chain, which can be associated with an edge-state mode, is only visible when, again, the gaps between the metal discs disappear and the individual resonators merge into a monolithic structure. At a distance of 17 nm between the metal discs and thus with an actual SSH chain of individual resonators, a scattering signal that is localized at the chain’s ends could not be detected because of the low coupling strength and the associated spectral overlap of mid-gap mode and band-edge modes. Furthermore, the optical diffraction limit did not allow a detailed characterization of mode structures. Near-field microscopy of short zigzag chains was also reported (*13*). However, here, too, the coupling strength was limited because of a large metal disc distance of around 100 nm that does not allow to separate purported mid-gap modes and other modes. An indication of the outstanding feature of the nontrivial topological phase, i.e., an alternating light intensity from disc to disc that decreases exponentially into the chain bulk (*14*), could not be demonstrated. In addition, the fact that the operation of zigzag chains requires polarized light forbids to extend the concept beyond 1D systems. Further studies of plasmonic SSH systems involved parallel linear waveguide systems in which the interwaveguide couplings alternate (*15*). While these systems have proven to be interesting platforms to demonstrate SSH functionality at mid-infrared frequencies, they lack deep subwavelength field confinement at optical frequencies to enable strong coupling with quantum emitters.

**Fig. 1.**
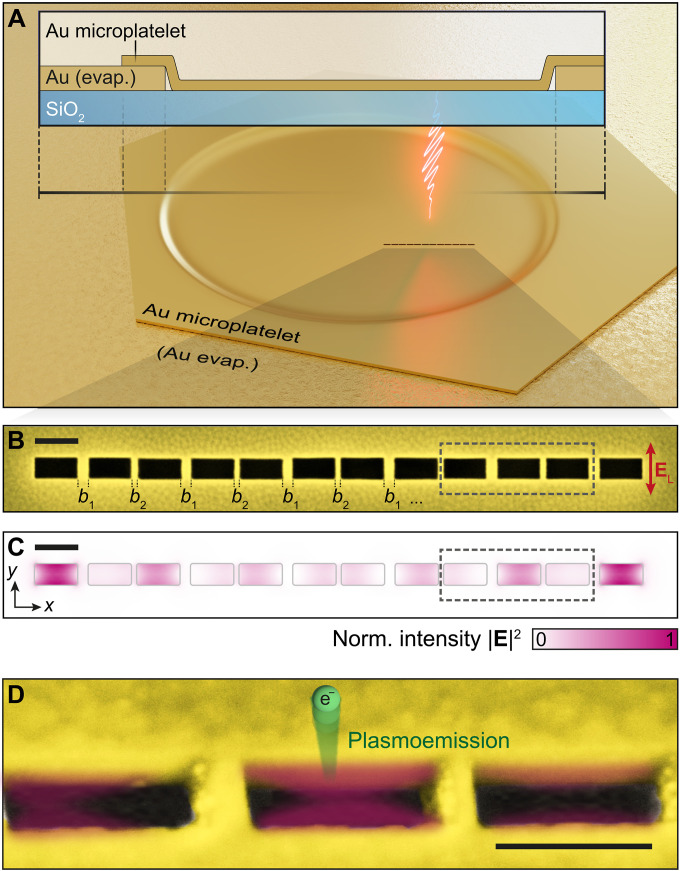
Plasmonic nanoslit SSH chain with nontrivial topology: Sample preparation, detection, and topologically protected mid-gap mode. (**A**) Chains of plasmonic nanoslit resonators written into a monocrystalline Au microplatelet using He-FIB. The microplatelet covers a hole in a vapor-deposited Au film created by a shadow mask. The nanoslit resonators therefore reside directly on a smooth glass substrate, while electric conductivity for PEEM is provided. Wide-field excitation (~270 μm in spot diameter, much larger than a single SSH chain, flat wavefront) with ultrashort laser pulses leads to local plasmoemission of electrons according to the mode pattern that is imaged with PEEM. (**B**) Top-view scanning electron microscopy (SEM) image (false color) of a nontrivial nanoslit SSH chain consisting of 12 coupled resonators separated by bridges with alternating widths, $b_{1}$ and $b_{2}$, as indicated. The polarization of the exciting electric field **E**_L_ [see artistic pulse in (A)] is oriented along the short axis of the nanoslits. (**C**) Simulated near-field intensity of a mid-gap mode (COMSOL) exhibiting localized near-field intensity at the two outermost nanoslits. (**D**) Side-view close-up SEM image of three nanoslit resonators, marked with a dashed rectangle in (B) and (C), highlighting the quality of the structures. A purple overlay indicates the local near-field intensity distribution. An electron released via plasmoemission is indicated in green. All scale bars represent a length of 100 nm. 3D rendering is shown in (A). Credit: T. Feichtner.

Here, we advance plasmonic topological SSH chains by mapping their subwavelength-localized, topologically protected mid-gap mode under rather strong coupling conditions of individual chain links. Using photoemission electron microscopy (PEEM), we achieve nanoscale-resolved imaging of plasmonic modes, revealing how the modal structure depends on the system’s geometry, i.e., its topological configuration (Fig. 1). The SSH chains consist of coupled Babinet-type nanoslit resonators (Fig. 1B), which allows the structure to be integrated and addressed in plasmonic circuits via plasmonic slot waveguides (*16*) or channel plasmon waveguides (*17*) with a minimum of material processing. Precise control over their spacing provides a large contrast between the alternating coupling strengths while always ensuring strong coupling to open up a sufficiently large energy gap. The strong coupling of the nanoresonators, and thus also the large spectral separation of topologically protected mid-gap modes from band-edge modes, allows us to experimentally confirm the presence of edge-localized modes in the middle of the gap by their distinct mode patterns. Furthermore, this spectral separation also opens up the possibility to specifically couple the topologically protected mid-gap modes to excitonic systems across material platforms. Because of the strong coupling of the nanoresonators within the SSH chain, this study represents a decisive milestone in the control of topological states in the subwavelength range and, due to the extensibility of the structures to two spatial dimensions, also lays the foundation for future investigations of higher-order topological plasmonic systems (*18*), their coupling to quantum emitters (*19*), and their potential integration into quantum photonic technologies.

## RESULTS

### Sample design and preparation

As fundamental building blocks of our plasmonic SSH chains, we use plasmonic nanoslit resonators (*20*, *21*), the Babinet counterpart of well-established nanorod dipole antennas (*22*). This choice is particularly suited to meet the requirements of PEEM: At the light intensities required for photoelectron generation via multiphoton absorption, isolated nanostructures, such as nanorods, are prone to structural damage, and heat generated by absorption cannot be efficiently dissipated. Moreover, sufficient sample conductivity is essential to prevent charging effects that cause electromigration and discharges. By fabricating the nanoslits in a monocrystalline Au microplatelet directly connected to a vapor-deposited Au film (Fig. 1A), we achieve enhanced conductivity for both heat and charge carriers compared to isolated structures on conductive oxides such as indium tin oxide. In addition, the use of nanoslits simplifies the manufacturing process, as only the material for the nanoslits needs to be removed and offers the possibility of integrating the SSH chain into plasmonic circuits via slot or channel plasmon waveguides. To achieve the required level of accuracy, we apply helium-focused ion beam milling (He-FIB; helium ion microscope, ZEISS ORION NanoFab) to monocrystalline Au microplatelets. The material’s monocrystallinity ensures constant milling rates everywhere on the platelet. As a result, high fabrication accuracy and reproducibility can be obtained.

**Fig. 2.**
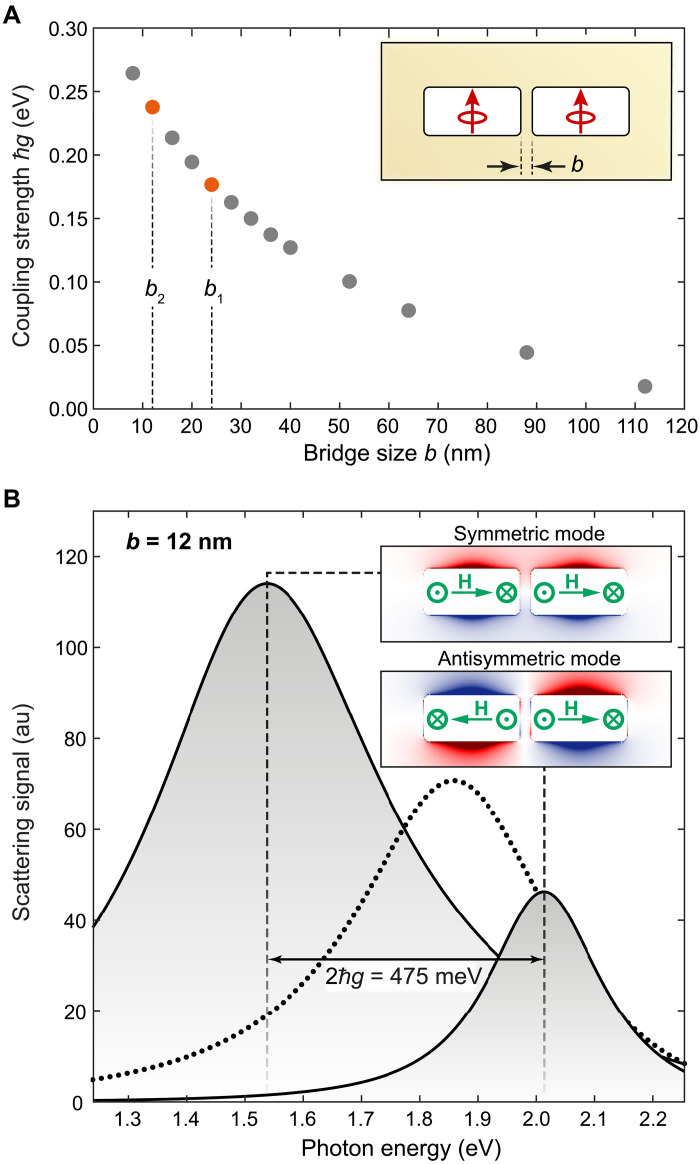
Coupling properties of a nanoslit dimer as retrieved from FDTD simulations. (**A**) Coupling strength as a function of the distance *b* between the nanoslits. As indicated in the inset, two electric dipoles, one positioned in the center of each nanoslit resonator, serve as the source to selectively excite the symmetric or antisymmetric hybridized eigenmode by setting the dipoles’ relative phase to 0 or π, respectively. In both cases, a transmission box monitor surrounding the structure measures the outgoing energy as a function of the oscillation frequency of the dipoles, resulting in a resonance curve. The value ℏg of the coupling strength is calculated via the spectral distance ΔE of the peak positions of the symmetric and antisymmetric hybridized eigenmode, i.e., ΔE/2=ℏg. The orange data points mark the coupling strength expected for nanoslit distances $b_{1}=24\;\text{nm}$ and $b_{2}=12\;\text{nm}$ of the fabricated structures. (**B**) Resonance curves of the symmetric and antisymmetric eigenmode for a bridge size of *b* = 12 nm (solid curves) and the monomer resonance curve (dotted line). The corresponding charge distributions of the dimeric eigenmodes (inset) were determined with built-in monitors and scripts of the commercial FDTD software. Green arrows mark the predominant direction of the magnetic field **H**. This field results for each monomer from the electric current flowing along the edges, driven by the electric field that can be represented by two magnetic dipoles, symbols ⊙ and ⊗, which are aligned perpendicular to the figure plane and exhibit a phase difference of π. au, arbitrary units.

Fabricated SSH chains consist of 12 nanoslits (Fig. 1B) separated by alternating bridges of $b_{1}=24\;\text{nm}$ and $b_{2}=12\;\text{nm}$ in width, with an SD of below 1 nm (see Supplementary Text and fig. S3). The nanoslits, resonant at the excitation laser wavelength of about $\lambda_{L}=680\;\text{nm}$ (Fig. 2B), were fabricated with a width of w=50 nm and a length of L=100 nm (Fig. 1B). The total depth of the nanoslits is determined by the thickness of the monocrystalline Au microplatelet and the incision depth in the glass substrate, which is caused by milling into the glass substrate after cutting through the gold microplatelet. The thickness of the monocrystalline Au microplatelet near its edge was measured with an atomic force microscope to be around 42 nm and is expected to be a few nanometers smaller at the nanoslit positions due to global material removal by the He-FIB process (more information in the Supplementary Materials). To produce stable nanometer-sized bridges, we applied parallel He-FIB structuring of the entire SSH chain. Please refer to the Supplementary Materials for detailed information about the manufacturing process.

### Strong coupling of nanoslit dimers

A prerequisite for the design of nanoslit SSH chains is the characterization of the coupling properties of end-to-end aligned nanoslit resonator pairs depending on the bridge size b. To this end, we carry out finite-difference time-domain (FDTD) simulations. The value of coupling strength ℏg (Fig. 2A) is determined by the spectral splitting ΔE=2ℏg of the two resulting nanoslit dimer eigenmodes, where the associated resonance curves were determined by measuring the output energy as a function of the oscillation frequency of the dipoles using a transmission box monitor surrounding the entire structure. To accurately determine the spectral position of the two eigenmodes, which can be difficult because of spectral overlap, we perform two separate simulations in which the excitation conditions were chosen, such that only one mode is selectively excited (Fig. 2B). Two electric dipoles serve as sources, with one dipole positioned in each nanoslit resonator and oriented along the short axis of the respective resonator (Fig. 2A, inset). Note that because of the Babinet principle, the far-field excitation of a dipole-like resonance, i.e., the lowest-order cavity mode of a single nanoslit antenna, requires an electric field polarization along the short axis of the resonator (*23*). The selection of the eigenmodes was then achieved by setting the relative phase between the dipoles to either 0 or π.

In Fig. 2A, the coupling strength ℏg extracted from a series of these simulations is displayed as a function of the bridge size b. As expected for the coupling of plasmonic nanoresonators, the coupling strength increases nonlinearly with decreasing bridge size. For a bridge size b<10 nm, the coupling strength reaches values of more than 250 meV. The data points marked in orange (Fig. 2A) indicate a coupling strength of 177 and 238 meV for bridge sizes $b_{1}=24\;\text{nm}$ and $b_{2}=12\;\text{nm}$, respectively, which were used to fabricate plasmonic SSH chains. We note that these coupling strength values are close to or even higher than 10% of the single nanoslit resonance energy of about 1.859 eV (667 nm) and thus fall into the regime of ultrastrong coupling (*24*). Here, we point out that, in contrast to the “strong coupling” regime, the “ultrastrong coupling” regime is defined by the ratio of the coupling strength to the bare excitation energies and not by the relationship to the system losses (*24*). For a more detailed discussion of coupling regimes, we refer to Supplementary Text. Another observation is that the coupling strength decreases by more than one order of magnitude for a bridge size b>100 nm compared to the largest bridge size $b_{1}$ of our SSH chain. We thus exclude an appreciable contribution from next-nearest-neighbor coupling in our SSH chain design in which the next-nearest nanoslit is located at a distance of $L+b_{1}+b_{2}=136\;\text{nm}$. This finding is relevant since the original SSH model (*3*) is based exclusively on nearest-neighbor coupling. It should also be noted that the coupling strength of plasmonic nanoantennas depends on the spatial overlap of charge distributions and currents of the monomeric entities and is therefore also influenced by the nanoslit width w. However, since the width of the resonators does not vary within SSH chains, we refer to Supplementary Text (pp. 12–13 and fig. S11) for more details.

The simulated characteristic charge distributions of the two nanoslit dimer eigenmodes for a bridge of $b=b_{2}=12\;\text{nm}$ are shown as an inset in Fig. 2B. Both eigenmodes exhibit charge accumulation along the short nanoslit axis. While charges of same sign accumulate on one side of the nanoslit dimer for the eigenmode at 1.537 eV (807 nm), charges of different sign accumulate on the same side of the nanoslit dimer for the eigenmode at 2.013 eV (616 nm). Consequently, only the red-shifted symmetric eigenmode can be efficiently addressed by far-field excitation, with the polarization of the incoming electric field along the short axis of the nanoslit resonators, whereas the blue-shifted antisymmetric mode can hardly be excited from the far field using plane waves at normal incidence. This behavior is in contrast to H-type coupling, i.e., the head-to-head configuration of electronic dipoles, in the context of molecular excitons. In excitonic H-type coupling, the energy of the bright transition is blue-shifted, similar to conventional plasmonic nanorod dimers where the individual nanorods, and hence the electric dipole moments of the associated eigenmodes, are aligned with their long axis parallel to each other. The energetic position of hybridized plasmonic modes in relation to each other can often be deduced from the Coulomb interaction within the characteristic charge distribution. In the case of Babinet nanoresonators, however, it must be remembered that the interaction of magnetic dipoles plays an important role in the coupling of eigenmodes (*25*–*27*). The electric currents that flow along the edges of a nanoslit monomer can be described by two out-of-plane magnetic dipoles that exhibit a π-phase shift, i.e., the overall current flow creates a magnetic quadrupole with an effective magnetic field **H** oriented along the nanoslit’s long axis (Fig. 2B, inset). For the symmetric lower energy eigenmode, the magnetic dipole moments at the dimer bridge are aligned antiparallel to each other, whereas in the case of the antisymmetric higher-energy mode, two magnetic dipoles are aligned parallel to each other. Therefore, the repulsion or attraction of magnetic poles in Babinet-type nanoslit resonators seems to take over the role of the Coulomb interaction of electric charges in conventional plasmonic nanoresonators when it comes to predicting the energetic position of hybridized eigenmodes.

### From nanoslit dimers to chains with topological features

When composing topological systems in plasmonics, special attention must be paid to the coupling range between monomeric entities. Long-range interactions can not only influence the eigenenergies of the chain modes, for example, but also lead to topological phase transitions. A system that is in the nontrivial phase, with respect to nearest neighbor coupling, can be transferred to the trivial phase by sufficiently strong long-range interactions (*14*).

To show that the plasmonic SSH chain (Fig. 1B) with an H-type electric dipole configuration is in the topologically nontrivial phase, we will prove the existence of the associated mid-gap modes by a simulation-based mode decomposition. For this purpose, we will compare the plasmonic eigenmodes with the quantum mechanical eigenstates of a typical SSH Hamiltonian. We start with the quantum model and represent our system of 12 nanoslit resonators via a tight-binding Hamiltonian $\hat{H}$ of 12 equivalent two-level systems with only nearest neighbor coupling

$$\hat{H}=\sum_{i=1}^{12}E_{0}{\hat{\sigma }}_{i}^{+}{\hat{\sigma }}_{i}+\sum_{\begin{array}{c} i=1\\ i\;\text{odd} \end{array}}^{11}v\left({\hat{\sigma }}_{i}^{+}{\hat{\sigma }}_{i+1}+{\hat{\sigma }}_{i}{\hat{\sigma }}_{i+1}^{+}\right)+\sum_{\begin{array}{c} i=2\\ i\;\text{even} \end{array}}^{10}w\left({\hat{\sigma }}_{i}^{+}{\hat{\sigma }}_{i+1}+{\hat{\sigma }}_{i}{\hat{\sigma }}_{i+1}^{+}\right)$$ (1)

where $E_{0}$ is the monomer resonance energy and v and w represent the values for the alternating coupling strengths between different monomer sites in units of energy. For each of the degenerate resonators within the chain, ${\hat{\sigma }}_{i}^{+}$ and ${\hat{\sigma }}_{i}$ are the associated fermionic creation and annihilation operators, respectively, considering only single excitations. In this limit, the diagonalization of the Hamiltonian (Eq. 1), which consists of 12 equivalent monomers, results in a common ground state and 12 new eigenstates in the manifold of single excitations. This Hamiltonian corresponds to that of the original work (*3*) of Su *et al.*, except for the missing spin degree of freedom, the missing interaction details of hydrogen and carbon atoms in polyacetylene, and the first term, which serves as a gauge for the energy scale.

**Fig. 3.**
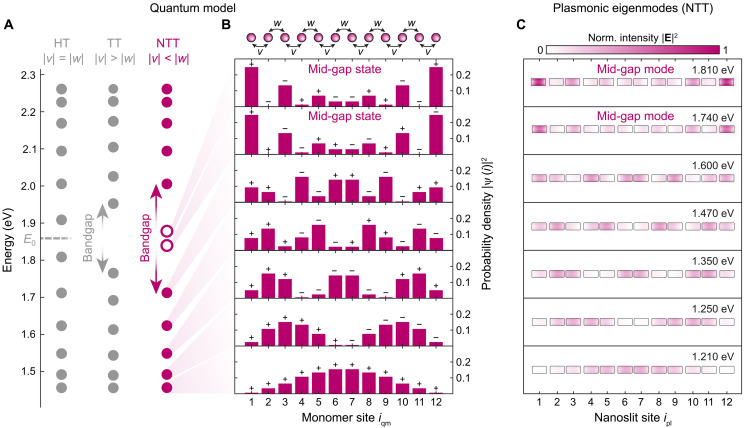
Modeling the nontrivial topological phase of the plasmonic nanoslit chain. Comparison of the mode patterns of a quantum mechanical SSH chain (see Eq. 1) consisting of equivalent two-level systems in the nontrivial topological phase to the corresponding mode patterns obtained via a quasi-normal mode analysis. (**A**) Eigenenergies of the single-excitation manifold of 12 coupled resonators with equivalent excitation energies of $E_{0}=1.851\;\text{eV}$ (gray dashed line). HT (v=w=−207 meV), TT (v=−238 meV and w=−177 meV), and NTT (v=−177 meV and w=−238 meV) with coupling amplitudes v and w. In the NTT case, the unfilled circles mark the odd and even parity mid-gap mode. (**B**) Probability density of the lower band and edge-state eigenfunctions of the quantum mechanical SSH chain in NTT configuration. The representation in the monomer site basis illustrates the localization of the excitations. (**C**) Near-field intensity distribution of the plasmonic nanoslit chain, at half of its height, in NTT configuration ($b_{1}=24\;\text{nm}$, i.e., 177-meV coupling strength, and $b_{2}=12\;\text{nm}$, i.e., 238-meV coupling strength) for the seven lowest eigenmodes obtained by quasi-normal mode analysis (COMSOL).

The systematics of the energy distribution of eigenstates in the manifold of single excitations crucially depends on the ratio of the coupling constants v and w. For uniform coupling v=w, the so-called homogeneous topology (HT), a homogeneous distribution of energy eigenvalues around the monomer energy $E_{0}$ is obtained (Fig. 3A), which would be gapless in the limit of infinite monomer sites. Note that only few states in the excited-state manifold can be excited efficiently from the ground state due to the oscillator strength redistribution (*28*). In the case of excitonic H-type aggregates, these are the states of highest energy. Breaking the translational symmetry of the coupling constant via staggering, i.e., v≠w, results in the splitting of the energy spectrum into two distinct bands separated by a gap (Fig. 3A). In addition, two topologically different phases can be distinguished (*2*): A phase of trivial topology (TT) for ∣v∣>∣w∣ and of non-TT (NTT) for ∣v∣<∣w∣.

Figure 3A shows explicitly the energy spectrum for the TT phase with v=−238 meV and w=−177 meV and for the NTT phase with v=−177 meV and w=−238 meV. Note that the values for v and w correspond to the coupling strength of a plasmonic nanoslit dimer for bridge sizes of b=12 nm and b=24 nm (see Fig. 2A). The negative sign accounts for H-type coupling of electric dipoles in the case of plasmonic systems. Moreover, the monomer energy is set to $E_{0}=1.859\;\text{eV}$, i.e., the resonance energy of a single plasmonic nanoslit resonator (Fig. 2B). As compared to the TT phase, in the NTT phase, the gap opens and two states appear in the middle of the bandgap (unfilled circles in Fig. 3A). These are the edge states, also called mid-gap states, which are topologically protected against perturbations, e.g., structural deformation.

In Fig. 3B, we show the probability density ${|\psi \left(i_{\text{qm}}\right)|}^{2}$ of having a single excitation in the NTT phase at the position of the *i*th monomer. More precisely, $\psi \left(i_{\text{qm}}\right)=\sum_{i}|i\rangle \left\langle i|\psi \right\rangle$ is the wave function of an SSH chain eigenstate ∣ψ⟩ expressed in the site basis $\sum_{i}|i\rangle \langle i|$, where $|i\rangle =|\ldots ,g_{i-1},e_{i},g_{i+1},\ldots \rangle$ describes the excitation of the ith monomer. While the eigenstates in the lower (and upper) band are delocalized over the entire chain, the mid-gap states are strongly localized at the outer two monomers, $i_{\text{qm}}=1$ and $i_{\text{qm}}=12$ (see figs. S6 and S7 for all wave functions of the NTT and TT configuration). The localization can be increased by increasing the ratio w/v since the probability density of these states decreases exponentially into the chain with a characteristic decay length of $\xi =\frac{1}{\left|\text{log}\left(v/w\right)\right|}$, which is measured in number of sites (*2*). Although the mid-gap states are completely identical in terms of the probability density ${|\psi \left(i_{\text{qm}}\right)|}^{2}$, they exhibit opposite parity: The sign of the real part of the monomer-resolved probability amplitude $\psi \left(i_{\text{qm}}\right)$ is denoted by + and − signs in Fig. 3B (see figs. S6 and S7). As a result, the higher-energy mid-gap state has an even parity, while the lower-energy mid-gap state has an odd parity. Consequently, only the higher-energy mid-gap state can be excited from the far field using a plane wave at normal incidence. Because of the relatively short length of the chain, the two mid-gap states are the symmetric and antisymmetric superpositions of the real edge states. In the case of a very long chain, these real edge states would be localized either on the left or on the right side of the chain and would be degenerate in energy. Hence, the different parity of the mid-gap states is a consequence of the symmetric and antisymmetric superpositions of the left- and right-localized edge states. We also point out that the parity of the mid-gap states swaps when an additional dimer is introduced into the finite chain. For each additional dimer, a new eigenstate is then added in the lower band so that the order of parity for the mid-gap states changes accordingly.

To verify that exclusive nearest-neighbor coupling is a valid assumption, we now compare the probability density ${|\psi \left(i_{\text{qm}}\right)|}^{2}$ of the SSH chain in the NTT phase with the quasi-normal modes of the fabricated nanoslit resonator chain determined using COMSOL (Fig. 1B and see Materials and Methods for details). According to the distance-dependent coupling strength (Fig. 2A), the staggered nanoslit bridges $b_{1}$ and $b_{2}$ should result in a nontrivial topological phase of the plasmonic system since $|v\left(b_{1}\right)|<|w\left(b_{2}\right)|$. As expected from the hybridization of 12 individual resonator modes, we find 12 eigenmodes for the nanoslit chain, of which we show the spatially resolved near-field intensity for the seven lowest-energy modes in Fig. 3C (see fig. S8 for all modes). The agreement of the near-field intensity distribution obtained by solving Maxwell’s equations and the probability density of the quantum model assuming the nearest-neighbor hopping of single excitations (Fig. 3B) is notable: Even if the plasmonic system does not consist of point-like chain elements but extended spatial structures with the corresponding spatially extended modes, the progression of the field intensity from nanoslit to nanoslit corresponds exactly to the progression of ${|\psi \left(i_{\text{qm}}\right)|}^{2}$ confirming the validity of the pure nearest-neighbor coupling assumption.

Note that there is a slight difference between the eigenenergies of the plasmonic modes compared to the energies in the single-exciton manifold. Such a deviation is expected since the spectral splitting due to the hybridization of plasmonic modes is not symmetric with respect to the resonance energy of the individual nanoslit and is not considered in Eq. 1. Asymmetric splitting occurs because of the nonlinear Coulomb interaction between the electron densities involved (*22*). For the present plasmonic system, the eigenenergies of the lower and upper band-edge states, as obtained from the mode decomposition, amount to E=1.600 eV and E=1.920 eV, respectively, resulting in a bandgap of about $E_{\text{gap}}=320\;\text{meV}$ (see the Supplementary Materials for the spectral data of all modes). Expressed in relative scales, the size of the bandgap is larger than 15% of the excitation energy of the nanoslit resonator.

Mid-gap modes in the fabricated nanoslit chains are expected to be found at eigenenergies of 1.740 and 1.810 eV, red-shifted with respect to the resonance energy of the individual nanoslit resonator (Fig. 3C). Note that a red shift of both mid-gap modes with respect to the resonance frequency of the individual resonator was also observed in a theoretical investigation of nanoparticle SSH chains (*14*). The alternation from high to low electric field intensity from nanoslit to nanoslit, which is archetypal for these states (*14*), can be recognized together with the exponential overall decrease in the intensity into the bulk of the chain. We therefore conclude that the fabricated plasmonic system is expected to be in the NTT configuration. Furthermore, we point out that, according to the correspondence of ${|\psi \left(i_{\text{qm}}\right)|}^{2}$ and ${|E\left(r\right)|}^{2}$, the localization of electromagnetic energy can be increased at the outer nanoslits by increasing the ratio $w\left(b_{2}\right)/v\left(b_{1}\right)$, i.e., increasing the bridge size of $b_{1}$ compared to $b_{2}$. However, we intendedly avoid this regime since the extreme case of a large ratio $b_{1}/b_{2}$ simply results in a system of isolated nanoslit dimers and single nanoslit resonators with an unperturbed resonance energy at each end (*10*).

### PEEM imaging of edge-state modes

To experimentally prove the existence of topologically protected plasmonic edge-state modes in He-FIB–fabricated SSH chains, we combine aberration-corrected PEEM, featuring a spatial resolution of about 3 nm (*29*), with a broadband noncollinear optical parametric amplifier (NOPA) system (full width at 10th maximum Δλ=120 nm or ΔE=200 meV at $\lambda_{L}=675\;\text{nm}$ or $E_{L}=1.837\;\text{eV}$, respectively) as excitation source to measure the mode structure shown in Fig. 3C. In PEEM, the dependence of the instantaneous photoelectron yield Y(r,t) on the *N*th power of local intensity ${|E_{\text{loc}}\left(r,t\right)|}^{2N}$ of the electric near-field of the plasmons is used as imaging contrast that enables deep-subwavelength spatial and femtosecond temporal resolution (*30*). Note that N is the number of absorbed photons so that the imaging contrast for plasmonic excitations in the optical spectral range is based on nonlinear photoemission. Davis and coworkers (*31*) had shown that it is even possible to reconstruct the full vectorial electric field for surface plasmon polaritons with PEEM. However, since we are only interested in the energy density distribution of plasmonic modes within the SSH chain, the intensity-dependent scalar value for the time-integrated photoelectron yield $Y\left(r\right)\propto \int_{-\infty }^{\infty }{|E_{\text{loc}}\left(r,t\right)|}^{2N}\mathrm{dt}$, i.e., a static PEEM image, is sufficient.

**Fig. 4.**
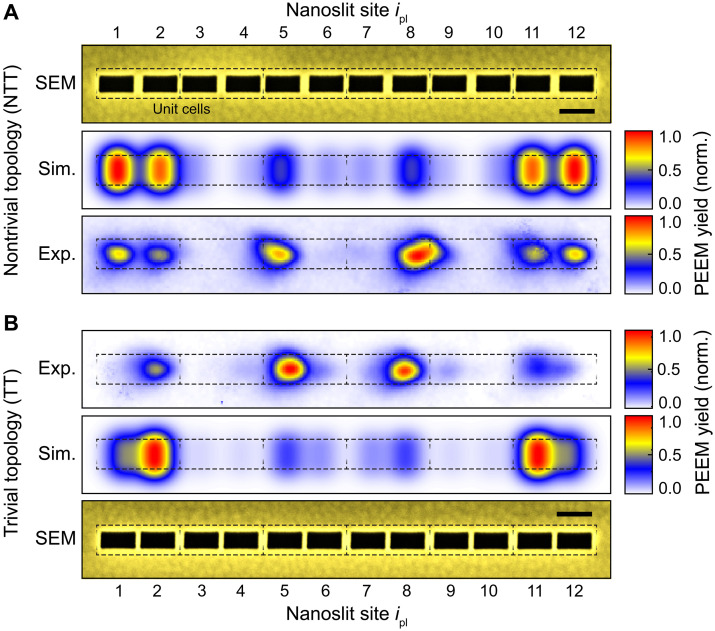
Imaging of the near-field intensity distribution of the plasmonic edge-state mode via PEEM. (**A**) Plasmonic nanoslit chain in the nontrivial configuration ($b_{1}=24\;\text{nm}$ and $b_{2}=12\;\text{nm}$). Top: SEM image. Simulated (middle) and measured (bottom) spatially resolved photoelectron emission yield upon pulsed laser irradiation (normal incidence, $\lambda_{0}=675\;\text{nm}\;\hat{=}\;1.837\;\text{eV}$). The nonlinearity of the photoemission process is N=3 (see the Supplementary Materials and fig. S10). The vectorial field data of FDTD simulations are acquired from a planar monitor, located 3 nm above the surface of the Au microplatelet, with a uniform mesh of 1 nm. See Materials and Methods for details. (**B**) Plasmonic nanoslit chain in the trivial configuration ($b_{1}=12\;\text{nm}$ and $b_{2}=24\;\text{nm}$). Measured (top) and simulated (middle) spatially resolved photoelectron emission yield upon pulsed laser irradiation at normal incidence. Bottom: SEM image. Scale bars, 100 nm. Dashed rectangles indicate the unit cells of the SSH chains.

Figure 4A (Exp.) shows a typical PEEM image of an SSH chain that was fabricated using the parameters employed in the COMSOL simulations (Fig. 3C; nanoslit length L=100 nm, width w=50 nm, and bridge sizes $b_{1}=24\;\text{nm}$ and $b_{2}=12\;\text{nm}$; i.e., the chain is expected to be in the NTT phase). This is also visualized by the dashed rectangles indicating the unit cells of the chain and revealing a larger intracell than intercell distance of the resonators. By comparing the scanning electron microscopy (SEM) image (Fig. 4, SEM) to the recorded PEEM yield map, the hotspots recorded in PEEM can be assigned to individual nanoslit resonators. It is found that the signal strength at the resonators varies over the chain. In particular, signals are very weak at the two central resonators $i_{\text{pl}}=6$ and $i_{\text{pl}}=7$. In contrast, there is strong photoelectron yield at the outermost resonators $i_{\text{pl}}=1$ and $i_{\text{pl}}=12$. The distribution of the photoelectron hotspots appears to be mirror symmetric with respect to the chain center. Small differences in PEEM yield between resonators on mirrored positions can be explained by structural deviations that can lead to enhanced local fields, which, in turn, are translated into significant signal differences due to the nonlinearity of the photoemission process. In addition, small variations in the work function may play a role and residual cross-talk with nearby chains (minimum distance, ~1.5 μm). Nevertheless, the characteristic mirror-symmetric hotspot-like photoelectron emission pattern with maxima located at individual resonators suggests the excitation of a distinct collective plasmonic mode.

In Fig. 4B (Exp.), we show a typical PEEM image of a chain with the same nanoresonators (L=100 nm and w=50 nm) but a bridge-size sequence resulting in the TT phase ($b_{1}=12\;\text{nm}$ and $b_{2}=24\;\text{nm}$). The indicated unit cells show a correspondingly larger intercell distance between the nanoresonators (Fig. 4B, SEM). As for the NTT chain, we observe hotspots in the emission of photoelectrons attributable to specific resonator positions and an overall mirror symmetry of that pattern. Notably, the hotspots on the two outermost resonators of the TT chain, i.e., $i_{\text{pl}}=1$ and $i_{\text{pl}}=12$, are very weak compared to the bulk hotspots of the same chain, while the hotspots of the outermost resonators of the NTT chain are clearly visible and stand out compared to the overall bulk signal. This indicates that the PEEM image of the NTT chain reflects a mid-gap mode whose intensity is localized mainly at the two outer resonators.

To ensure that the visibility and brightness of the hotspots at the outer ends of the NTT chain compared to the TT chain are due to a plasmonic mode and not due to local defects in combination with the relatively large dynamic signal range associated with nonlinear photoelectron emission (N=3 in this case; see Materials and Methods, Supplementary Text, and fig. S10), we simulated the PEEM image signal Y(r) using the vectorial local response function R(x,y,z,ω) as retrieved from a planar monitor in FDTD simulations 3 nm above the Au microplatelet surface and the measured laser spectrum $E_{L}\left(\omega \right)$ (see Materials and Methods for details). The simulated PEEM images are displayed in Fig. 4 (A and B, Sim.) for the NTT and TT chain, respectively. The hotspot patterns in the simulated PEEM images match the pattern in the measured images quite well, particularly with respect to their position. The simulations confirm that the hotspots at the outermost nanoslit resonators $i_{\text{pl}}=1$ and $i_{\text{pl}}=12$ of the NTT chain, which are clearly visible in the PEEM experiment, can be assigned to the energetically higher lying mid-gap mode. This mid-gap mode, which matches the spectral window of the laser and is accessible from far field due to its even parity, can be unambiguously identified in the FDTD response function (see fig. S5). This kind of mode is not present in the FDTD response function of the TT chain, and, consequently, only weak photoemission can be detected at the outer two nanoslit resonators of the TT chain, in both experiment and simulation.

Taking the typical structure of the eigenmode of the edge state (Fig. 3C, mode pattern at 1.810 eV) as the basis for the PEEM image contrast, however, the question arises as to why no alternating sequence of high and low photoemission signals from resonator to resonator can be observed in both the measured and simulated PEEM image of the NTT chain. The reason for this lies in the interplay of the topological invariant of the SSH chain, i.e., a geometric phase acquired over the Brillouin zone, which is called the Zak phase $\phi_{\text{Zak}}$ (*32*–*34*), and the excitation of the entire chain with a homogeneous wavefront and polarization direction of the incident field. In the NTT configuration of the SSH chain, the Zak phase takes the value $\phi_{\text{Zak}}=\pi$, while $\phi_{\text{Zak}}=0$ for the TT configuration. In our plasmonic system, $\phi_{\text{Zak}}=\pi$ signifies that the electromagnetic field distribution acquires a phase shift of π when moving across the boundaries of the unit cells (*14*, *35*), indicated by the dashed rectangles in Fig. 4A. That is, the electric field of the mid-gap mode changes its sign from resonator $i_{\text{pl}}=1$ to $i_{\text{pl}}=3$ to $i_{\text{pl}}=5$, and so on, just as the real part of the edge-state wave functions in the quantum model changes sign at the same nanoslit sites (Fig. 3B). Consequently, an incident plane wave with a given polarization direction would interfere destructively with the edge-state mode field at $i_{\text{pl}}=3$, for example, if the incident field interferes constructively with the edge-state mode field at nanoslit resonators $i_{\text{pl}}=1$ and $i_{\text{pl}}=5$ at the same time. Since wide-field excitation is used in both experiment and simulation, the Zak phase of the NTT chain is indicated by the absence of the photoemission signal at $i_{\text{pl}}=3$ and $i_{\text{pl}}=10$, while, at the same time, the photoemission signal at the outer two nanoslit resonators $i_{\text{pl}}=1$ and $i_{\text{pl}}=12$ is clearly recognizable. The archetypal look of the edge-state mode as seen in Fig. 3C is obtained in FDTD simulations if only the outer two nanoslit resonators $i_{\text{pl}}=1$ and $i_{\text{pl}}=12$ are selectively excited by spatially restricted plane-wave sources (see Supplementary Text and fig. S5). Therefore, considering the plane-wave excitation condition, the recorded hotspot pattern of photoemission of the NTT chain is indeed the expected signature of a topological edge-state mode.

Another interference effect explains that in the case of the NTT chain, the measured photoelectron yield at the inner nanoslit resonators $i_{\text{pl}}=5$ and $i_{\text{pl}}=8$ is slightly higher than at the outer nanoslit resonators $i_{\text{pl}}=1$ and $i_{\text{pl}}=12$, although the FDTD-supported simulation of a single NTT chain predicts an opposite behavior (Fig. 4A). In this case, the interference of the electromagnetic field of the edge-state mode with the field of propagating surface-plasmon polariton modes, injected at neighboring SSH chains upon wide-field excitation, causes a deviation from the local field as expected for an isolated NTT chain (see Supplementary Text and fig. S9). To measure SSH chains with different parameters within the same field of view in the PEEM, these chains cannot be produced arbitrarily far away from each other. As a consequence, the local field intensity, which is responsible for the photoelectron yield, can always slightly deviate from the electromagnetic field intensity of the pure edge-state mode.

## DISCUSSION

We have fabricated high-precision plasmonic SSH chains with a unit cell length of 236 nm from individual nanoslit resonators and were able to demonstrate and visualize the existence of archetypal edge states in the nontrivial configuration of the chain experimentally via PEEM revealing strong spatial field localization at the outermost nanoslits and the characteristic mode pattern of a mid-gap state. This behavior is theoretically predicted by a quantum model considering nearest-neighbor coupling and a matching quasi-normal mode analysis. By combining He-FIB and monocrystalline Au microplatelets as the structural material, we precisely realized the required nanoslit spacings down to 12 nm, with an SD smaller than 1 nm leading to coupling strengths between two nanoslit resonators in the ultrastrong coupling regime. This results in a bandgap of 320 meV for the entire SSH chain, which corresponds to more than 15% of the nanoslit resonance energy.

With the large bandgap obtained in this study, we have achieved spectral separation of a mid-gap mode from the band-edge modes, which is an important step to connect excitonic excitations beyond all material classes to these topologically protected plasmon modes. As observed from the scattering signal in Fig. 2B and the FDTD response function of the plasmonic SSH chain in NTT in fig. S5A, the spectral full width at half maximum (FWHM) of the individual nanoslit modes and the mid-gap mode, respectively, is in the range of ~200 meV. To further improve the spectral selectivity, i.e., to increase the ratio of the bandgap to spectral linewidths, we suggest reducing the width of the nanoslit resonators from w=50 nm to, e.g., w=20 nm. Some of us had shown experimentally that these narrow (open-end) nanoslit resonators can achieve a *Q* factor of 20 (*36*), which would lead, in our case, to a halving of the FWHM to ~100 meV at a mid-gap mode energy of $E_{0}=1.851\;\text{eV}$. A more detailed discussion about the impact of the nanoslit width *w* on the linewidth and coupling strength of nanoslit resonators can be found in Supplementary Text (pp. 12–13 and fig. S11).

The spectral separability of the mid-gap modes from the photonic bands is an important prerequisite for the coupling of excitonic excitations to topologically protected states of the system. In this scenario, the use of PEEM is particularly interesting, since the method enables the investigation of spatially resolved quantum dynamics of excitonic systems coupled to the SSH chain by means of nonlinear spectroscopy, i.e., 2D nanoscopy (*37*, *38*). In the case of plasmonic mode imaging, we were able to detect the edge-state mode and thus the presence of the nontrivial phase of the SSH chain by the combined influence of the associated Zak phase of $\phi_{\text{Zak}}=\pi$ and the PEEM-related wide-field excitation condition on the local near-field. As an interesting supplement to the wide-field excitation conditions in PEEM, cathodoluminescence spectroscopy (CLS) (*39*) offers the possibility of a highly localized, dipole-like excitation source. Since the emitted radiation is governed by the imaginary part of the electromagnetic Green’s function, CLS enables access to the spatial variation of the projected local density of states. In this context, CLS was used, for example, to map a valley-polarized plasmonic edge mode (*40*).

To conclude, our unambiguous experimental demonstration of the ability to control and spectrally isolate topological states in plasmonic systems, which exhibit subwavelength localization of plasmonic excitations in edge states, represents a decisive advancement in several ways. First, it paves the way for achieving strong coupling between single emitters and topologically protected edge states, enabling the exploration of fundamental questions about extending topological protection to hybrid quantum states of light and matter. Second, it opens new possibilities for the design of topological edge modes in 2D arrangements (*18*, *19*), despite their anisotropic shape (see fig. S12 and Supplementary Text for an assessment of the coupling strength in distinct lattice directions). This will enable advanced functionalities such as unidirectional energy transport with minimal loss, even stronger energy confinement in corner states, and the exploitation of long-range coherence between different corner states.

## MATERIALS AND METHODS

### PEEM setup

PEEM images were acquired using a Yb-doped fiber laser (Amplitude Systèmes, Tangerine HP; 1030 nm, 35 μJ, ~320 fs, 1-MHz repetition rate), which pumps a two-branch NOPA (Riedle group, LMU Munich) achieving a spectral range of Δλ=120 nm (full width at 10th maximum, corresponding to ΔE=200 meV) at the tailored central wavelength of $\lambda_{L}=675\;\text{nm}$ (or $E_{L}=1.837\;\text{eV}$). The pulses were compressed with a prism compressor and guided through a liquid crystal display–based spatial light modulator (Jenoptik, SLM-S640d USB) in a 4f geometry, enabling double-pulse scans. Pulse characterization was performed using spectral phase interferometry for direct electric field reconstruction setup (FC Spider VIS, APE Angewandte Physik und Elektronik GmbH).

The sample was irradiated under normal incidence with pulses of ~20-fs duration, a beam radius of $r_{x}=270\;\mu m$ and $r_{y}=405\;\mu m$, and 46-nJ pulse energy, preventing space-charge effects. The polarization of the laser pulses was set perpendicular to the SSH chains. Active beam stabilization (TEM Messtechnik, Aligna 4D) was used to correct for vibrational instabilities between laser setup and the separate PEEM table.

Photoelectrons emitted from a 5-μm-diameter field of view were collected using an aberration-corrected photoemission electron microscope (AC-LEEM III, Elmitec Elektronenmikroskopie GmbH), filtered in *k*-space via a 60-μm contrast aperture, and transferred to the detection unit. The detection unit consisted of two chevron-type microchannel plates for electron multiplication, a phosphor screen for electron-to-photon conversion, and a charge-coupled device camera for image acquisition.

We collected and analyzed both static images and double-pulse scans (pulse delay time τ=0 to 81 fs with a step size of δτ=0.3 fs) with an integration time of 90 s. The images from the double-pulse scan were integrated to obtain the static response after applying drift correction to compensate for small spatial drifts over the ~15-hour measurement time. Drift correction was performed using reference images acquired with single-pulse excitation and an integration time of 30 s. Through this procedure, we receive PEEM images with improved signal-to-noise ratio but the same mode pattern as in the static images. Further details on the experimental setup can be found elsewhere (*29*).

### Modeling PEEM yield with FDTD data

FDTD simulations considered a 700-nm-thick glass substrate and a 38-nm-thick Au layer describing a microplatelet. Material data were taken from Palik’s handbook (*41*) and from Johnson and Christy (*42*), respectively. The nanoslit resonators were modeled as rectangular holes with rounded corners and edges that exhibit a 6-nm radius of curvature. To take the He-FIB process into account, the nanoslit resonators extended 10 nm beyond the Au layer into the glass. The SSH chain was discretized using a mesh with a spatial resolution of 1 nm. To take the far-field excitation conditions into account, the SSH chain was homogeneously excited by plane-wave total-field scattered-field source, with a polarization perpendicular to the chain.

The FDTD response function R(x,y,z,ω) determined by these excitation conditions, i.e., the superposition of incoming field and excited plasmonic near-field, was recorded in a planar monitor 3 nm above the Au surface. We obtained the local near-field responsible for photoemission via $E_{\text{loc}}\left(x,y,z,t\right)=\text{FT}\left\{R\left(x,y,z,\omega \right)\cdot E_{L}\left(\omega \right)\right\}$, where $E_{L}\left(\omega \right)$ is the spectral amplitude of the measured laser spectrum and FT{…} denotes the Fourier transform. For modeling the PEEM yield according to the ansatz of the surface and volume photoelectric effect, we used the multiplasmon photoemission model of Podbiel and coworkers (*43*) in which the local field is split into a component perpendicular, i.e., ${E_{\text{loc}}}^{⏊}\left(x,y,z,t\right)=|E_{z,\text{loc}}\left(x,y,z,t\right)|$, and parallel, i.e., ${E_{\text{loc}}}^{\|}\left(x,y,z,t\right)=\sqrt{\;{\left|E_{x,\text{loc}}\left(x,y,z,t\right)\right|}^{2}+{\left|E_{y,\text{loc}}\left(x,y,z,t\right)\right|}^{2}}$, to the surface such that $Y\left(x,y,z\right)\propto \int_{-\infty }^{\infty }{\left\{{\left[{E_{\text{loc}}}^{\|}\left(x,y,z,t\right)\right]}^{2}+\alpha^{2}{\left[{E_{\text{loc}}}^{⏊}\left(x,y,z,t\right)\right]}^{2}\right\}}^{2N}\;\mathrm{dt}$, where N=3 is the number of absorbed photons, as determined experimentally (see Supplementary Text and fig. S10). The value of α=5.8 is an empirically found value (*43*) that not only is usually associated with a specific facet of the Au single crystal but also works reasonably well in our case, although it is not known which facets contribute to photoemission here. To model the PEEM images in Fig. 4, we evaluated Y(x,y,z) only at the points where gold is present as material in the lateral structural profile of the SSH chain. Last, we convoluted Y(x,y,z) with a 2D Gaussian that exhibits an FWHM of 70 nm to account for the reduced resolution of the photoemission hotspots near the nanostructured surfaces and any imaging errors in the PEEM alignment.

### Eigenmode decomposition with COMSOL

The structures and material properties used in our COMSOL simulations are identical to those in the FDTD simulations. A perfectly matched layer with a thickness of 250 nm was applied to suppress backward reflections at all boundaries of the simulation volume. Because of the mirror symmetry of the SSH chain structure with respect to the *xz* and *yz* planes, a refined mesh was required for only one-quarter of the entire structure, and the fields retrieved by mode analysis were accordingly mapped to the three residual domains. An eigenfrequency solver was used to search for all possible states within the system around the plasmonic nanoslit resonance, while filtering out any unphysical modes caused by numerical artifacts. Last, after postprocessing, we normalized all mode patterns to the maximum electric field intensity.

## Supplementary Materials

### This PDF file includes:

Supplementary Text Figs. S1 to S12 References
