# Supplementary material for: Plasmonic Su–Schrieffer–Heeger chains with strong coupling amplitudes
Source: Sci Adv. 2025 Dec 10;11(50):eaea3844. doi: 10.1126/sciadv.aea3844 (PMC12693959; doi:10.1126/sciadv.aea3844)
Supplement: Supplementary file 1 — Supplementary Text Figs. S1 to S12 References [file sciadv.aea3844_sm.pdf]

Science Advances
AAAS

# Supplementary Materials for

## Plasmonic Su–Schrieffer–Heeger chains with strong coupling amplitudes

Benedikt Schurr *et al.*

Corresponding author: Bert Hecht, bert.hecht@uni-wuerzburg.de; Tobias Brixner, tobias.brixner@uni-wuerzburg.de

*Sci. Adv.* **11**, eaea3844 (2025)
DOI: 10.1126/sciadv.aea3844

**This PDF file includes:**

Supplementary Text
Figs. S1 to S12
References

# Supplementary Text

## Fabrication of nanoslit SSH chains

Sample layout and determination of the thickness of the gold microplatelet

All Su–Schrieffer–Heeger (SSH) chains discussed in the manuscript were fabricated on a hole mask sample (Fig. S1A), specifically designed for photoemission electron microscopy (PEEM) experiments. For this, a 24 mm × 24 mm microscopy glass cover slip (cover glasses, Menzel Gläser) was used as a glass substrate which exhibits a thickness of 0.17 mm. Before processing, the glass substrate was thoroughly cleaned using a multi-step procedure. First, the glass substrate was placed in an ultrasonic bath with ultra-pure acetone and ethanol for 10–15 min. Afterwards, the glass substrate was rinsed by ultrapure water and blow-dried by pressurized nitrogen gas. Finally, remaining residues were removed by plasma cleaning (PlasmaFlecto 10, Plasma Technology) for 5 min at 250 W with an oxygen flow of 5 standard cubic centimetres per minute (sccm). Next, the glass substrates underwent an optical lithography process, during which they were coated with a 15 nm chromium layer followed by approximately 70 nm of thermally evaporated gold. An optical mask was used to obtain the desired patterns, e.g., marker structures, like a coordinate system. Specific sample positions were encoded by specific hole pattern matrices (see Fig. S1A,B). The circular shape of the holes prevents electrical charging at sharp corners during PEEM experiments. Furthermore, larger circular hole structures with diameters of up to 100 μm were fabricated on the sample using optical lithography. These large holes served as deposition sites for the monocrystalline gold microplatelets, into which the SSH chains were later written (Fig. S1C). Although gold exhibits higher optical losses in the visible spectral range than silver, we use self-grown monocrystalline gold microplatelets as a platform for nanoslit SSH chains. Gold's chemical stability and resistance to oxidation under ambient conditions make it a reliable and well-studied material, unlike silver. In addition, evaporated gold or silver films typically suffer from surface roughness and large grain sizes, which can cause disruptive PEEM hotspots and limit the nanometer precision of FIB milling. Monocrystalline silver platelets also present challenges: Lyutov and co-workers (*44*) reported a minimum thickness of about 100 nm, and even the improved value of about 70 nm achieved by Schörner and co-workers (*45*) is still

much thicker than the 40 nm accessible with gold. This thinner gold platform is essential for keeping the spectral resonances within the range of interest.

The synthesis of monocrystalline gold microplatelets with high aspect ratios is described in detail in Wu et al. (*46*) In the next step, an Au microplatelet was transferred from the growing substrate onto the hole mask sample, using an in-house developed technique by covering a microplatelet with a droplet of polymethylmethacrylate (PMMA). After baking the PMMA microplatelet composite at 100 °C for 1 h, the compound was picked up from the growing sample and placed onto the desired position on the hole mask. The PMMA was finally removed using an acetone bath.

After the transfer, the sample was again plasma-cleaned with the same specifications as before. In a final step, the hole mask sample was cut to a size of 1 cm × 1 cm using a diamond cutter tool to fit the sample holder of the PEEM.

The monocrystalline gold microplatelets are particularly suitable for nanofabrication using focused-ion-beam (FIB) milling. Here, we have used a helium ion microscope (Orion NanoFab, Zeiss) which combines a gallium (Ga) and a helium (He) FIB. While the Ga-FIB is commonly used to fabricate rather coarse and larger structures on short time scales, the He FIB can be used for fine structuring objects with nm precision. Therefore, Ga-FIB was used to create ring-like marker structures inside the microplatelet (Fig. S1C) to facilitate finding the desired position of the SSH-chains with PEEM while to fabricate sub–10 nm nanostructures (*47*). For the fabrication of nanoslits and SSH chains (Fig. S1D–F), the He-FIB was focused down to a spot size of below 1 nm and an acceleration voltage of 35 kV was used, providing an average ion current of 3.2–3.4 pA.

For the correct modelling of nanoslits and SSH chains, as well as for finding suitable patterning parameters for the fabrication procedure, the thickness of the gold microplatelet, i.e., the depth of the individual nanoslits, plays an essential role. Atomic force microscopy (AFM) was used to measure the thickness of the microplatelet, using a line-cut as depicted in Fig. S1G. As the AFM scan was performed at the very edge of the microplatelet, the maximum thickness

of the unstructured microplatelet is determined. The height profile and the corresponding histogram provide a thickness of the microplatelet of $(42.3 \pm 0.9)$ nm (Fig. S1H). Note that this value is valid at the edge of the microplatelet but, most likely, it is slightly smaller (a few nm) in the area where the fabrication is performed. This is due to the permanent removal of gold layers while scanning over the area of interest to find the preselected area. To reduce this effect, the number of He-FIB scans was reduced to a minimum before fabricating the final SSH chains.

Fabrication of nanoslit resonators using high-precision He FIB milling

In this section, the fabrication of the individual nanoslits and SSH chains is explained. For the fabrication of single nanoslits, rectangular FIB patterns were designed, and the corresponding FIB parameters were adjusted. Since the amount of gold milled from the microplatelet is highly dependent on the energy of the He ions, the ion current (rate), and the total sputtering duration, these parameters were carefully adjusted to achieve a precise and uniform cut of the predesigned rectangle into gold. Here, we have used a 35 kV He FIB with an average current of 3.4 pA that was focused onto the sample's surface. The decisive property of the FIB process is the He ion dose that is applied to the sample. Several parameters like the dwell time of the He-beam, or the number of FIB repeats determine the total dose. To determine the minimum dose required to completely cut through an approximately 40 nm thick gold microplatelet, dose tests were conducted. In Fig. S2A, a 3D AFM scan of a 3×3 nanoslit matrix is presented, showing different sputtering results for nine different dose values. A clear trend can be observed between a higher dose and a deeper incision into the gold. The doses for this test were varied between 0.1 $C/cm^2$ and 1.6 $C/cm^2$.

For a quantitative analysis of the relation between dose and FIB depth, the measured nanoslit depths are plotted versus the applied doses (Fig. S2B). A linear relationship is observed, showing an increase in nanoslit depth as a function of dose, with a rate of $-53.8\ (\mathrm{nm} \cdot \mathrm{cm}^2)/\mathrm{C}$. Furthermore, the last two data points corresponding to doses of 1.4 $C/cm^2$ and 1.6 $C/cm^2$, respectively, indicate a saturation of the achievable depth, which is attributed to the limited penetration depth of the AFM tip exhibiting a tip radius of ~10 nm. This is why the last two data points have been excluded from the evaluation. The insets in Fig. S2B depict line cuts (labelled

as A and B) from the AFM scan in Fig. S2A. While the bottom one shows a cut providing the width of the nanoslit, the inset at the top displays a line cut along three different nanoslits, fabricated with different He ion doses.

This line cut clearly shows that higher He ion doses lead to deeper nanoslits. Based on the dose tests and the fact that the gold microplatelet exhibits a maximum thickness of approximately 41 nm, a suitable dose for fabrication was determined to be between 1.0 C/cm² and 1.1 C/cm². This determined value ensures that the microplatelet is completely cut through while minimizing milling into the glass substrate. From Fig. S2B, the depth of milling into the glass was estimated to range between 12 nm and 15 nm. As previously discussed, the area of interest was scanned two to three times before fabrication for orientation and sample adjustments, so that a reduction of microplatelet thickness by a few nm is expected.

In Fig. S2C, a scanning electron microscope (SEM) scan of the same 3×3 nanoslit matrix from Fig. S2A is displayed. The overall trend and observations from the AFM investigation are confirmed. For low doses, a very low contrast is observed between the nanoslits and the surrounding, unstructured gold. On the one hand, for doses just below 1.0 C/cm² the SEM scan clearly shows that the He beam has nearly cut through the microplatelet, while some remaining gold can be observed inside the nanoslit. On the other hand, doses of 1.0 C/cm² and above indicate a full cut through the gold layers and an increasing sputtering of the glass substrate beneath. It should be noted that in the SEM scan, no significant differences could be observed for the nanoslits fabricated with He FIB doses of 1.2 C/cm² and higher since allegedly the contrast, generated by the scattered electrons at the glass substrate inside the nanoslit, remains approximately unchanged for doses ranging from 1.2 C/cm² to 1.6 C/cm². In Fig. S2D, another SEM scan of a 3×3 nanoslit matrix is depicted verifying the observations from the 50 nm × 100 nm nanoslits. In this case, the nanoslits were chosen to provide twice the aspect ratio of the nanoslits used for the actual SSH chains.

For the actual fabrication of the SSH chains, FIB patterns were designed to create a chain of twelve identical nanoslits, aligned in a row, and separated by nanoscale bridges of alternating bridge sizes $b_1$ and $b_2$. The widths of the bridges were designed to be 12 nm for the small bridges

and 24 nm for the large ones. Since a one-by-one fabrication of the individual nanoslits in an SSH chain inevitably led to the destruction of bridges, it was essential to change the fabrication technique towards a “parallel-patterning approach”. In this process, the individual nanoslits were produced simultaneously. Although the nanoslits were removed layer-by-layer, i.e., sequentially, the process restarted always at the same chain end after each pass, ensuring uniform material removal. Using this technique, it was possible to fabricate the delicate bridges between individual nanoslits.

Evaluation of bridge sizes of nanoslit SSH chains

The nanoscale bridges $b_1$ and $b_2$ separating the nanoslit resonators are the key parameters that determine the coupling between individual nanoslits. It is therefore important to precisely design, fabricate, and measure the widths of these bridges.

In Fig. S3A, a SEM scan of a nontrivial SSH chain is depicted (top panel), in which all bridges are labeled. Each fabricated SSH chain exhibits eleven bridges since it consists of twelve nanoslit resonators. For the case of nontrivial topology (NTT), the chain has six large bridges of width $b_1$ and five narrow bridges of width $b_2$. A statistical evaluation of all bridge widths of an NTT SSH chain was performed and bridge widths were measured directly after the fabrication process using SEM (GeminiSEM 450, Zeiss). In the bottom panel of Fig. S3A, a zoomed-in version of the image of the complete chain in the top-panel is shown. To quantify the bridge widths each bridge was measured along three different line-cuts, as indicated by the three blue lines.

The resulting bridge widths are plotted and analyzed in Fig. S3B. As is visible from the distribution of the data points, the bridge widths deviate only slightly from the designed bridge width of 24 nm and 12 nm. For the large bridges (black triangles), a mean value with an associated standard deviation of $\mathbf{(24.8 \pm 0.9)\ nm}$ is found, while for the small bridges (red dots), the mean value is found to be $\mathbf{(12.7 \pm 0.5)\ nm}$. The $\mathbf{1\sigma}$ standard deviation is indicated in the plot with gray and light-red rectangular stripes. This evaluation suggests that the fabrication precision is close to, or even below, 1 nm. In this fabrication approach, the bridge widths $b_1$ and $b_2$ are both slightly larger compared to the target values. This, however, does not

influence the overall topological behavior of the SSH chains and only slightly modifies the couplings.

In Fig. S3C,D we show two exemplary line cuts for the bridge width analysis. As indicated in the plots, the bridge width was determined by calculating the full width at half maximum (FWHM) for each line cut.

## Zak phase and its impact on near-field patterns

Zak phase influence on the electric field distribution of edge-state eigenmodes

In the main manuscript, we discuss that the absence of PEEM yield at nanoslit resonators $i_{\mathrm{pl}} = 3$ and $i_{\mathrm{pl}} = 10$ is due to the combination of the Zak phase and wide-field excitation conditions. To demonstrate that the Zak phase is present at all in the NTT configuration of the plasmonic SSH chain, we first show in Fig. S4 the $E_y$ component of the two mid-gap modes as obtained from our COMSOL eigenmode decomposition. We show here the $E_y$ component because the lateral field component perpendicular to the SSH chain corresponds to the resonant excitation condition of the nanoslit resonators. Indeed, for the mid-gap mode with even parity (Fig. S4A) the field amplitude at nanoslit resonators $i_{\mathrm{pl}} = 1,3,5$ exhibits an alternating sign, while the amplitude decreases into the bulk. According to the Zak phase of π, i.e., the change of signs in field amplitude across the unit cell, nanoslit resonators $i_{\mathrm{pl}} = 2$ and $i_{\mathrm{pl}} = 4$ exhibit electric field strength close to zero. Starting from the right end of the chain, the same amplitude progression is observable due to even parity. Note that one reason for a non-zero field amplitude at nanoslit resonators $i_{\mathrm{pl}} = 2,4,6$ and $i_{\mathrm{pl}} = 11,9,7$ are retardation effects in our finite-sized structures (*14*). Additionally, the total number of resonators, i.e., twelve, is so small that there is a significant overlap between the edge state at the left end of the chain and the edge state at the right end of the chain so that even within the quasi-static limit the mid-gap modes, resulting from hybridization of the two edge states, would also show a non-zero field amplitude at the respective nanoslit resonators. The odd-parity mid-gap mode (Fig. S4B) shows the same

behavior in the electric field distribution caused by the Zak phase, except that the signs of the fields on the right side of the chain are inverted with respect to the left side due to the odd parity.

Near-field patterns of the NTT SSH chain under different excitation conditions

Now that we have shown that the Zak phase of π, archetypal for the nontrivial phase of the SSH chain, is present in our plasmonic system, we consider the response function of the NTT chain under wide-field excitation, as it corresponds to the excitation conditions of the PEEM experiment. Our assumption is that the spatially alternating sign of the field strength within the mid-gap mode leads to both constructive and destructive interference with the homogeneous field polarization of the excitation source, and thus the photoemission yield does not reproduce the near-field distribution of the respective eigenmode one-to-one.

In Fig. S5A, we show the absolute square of the FDTD-retrieved response function under wide-field excitation along the chain axis, close to the edge of the nanoslit resonators as indicated by the horizontal light-blue line in the top panel. Even if this quantity, here called "intensity" for the sake of simplicity, cannot be taken directly as a measure for the photoemission yield (see the model for calculating the PEEM yield in the main manuscript), it does provide a qualitative impression of the photoemission yield. First, the high field intensity at the outermost nanoslit resonators, at an excitation energy of ~1.8 eV, clearly shows that the even-parity mid-gap mode lies well within the laser spectrum. But in contrast to the pure eigenmode (Fig. S5B), the field intensity at nanoslit resonators $i_{\mathrm{pl}} = 3$ and $i_{\mathrm{pl}} = 10$ is close to zero, which is consistent with the low photoemission yield in the experiment (Fig. 4a, Main Manuscript). We attribute this behavior to the change of sign in the electric field from the outermost resonators $i_{\mathrm{pl}} = 1$ and $i_{\mathrm{pl}} = 12$ to resonators $i_{\mathrm{pl}} = 3$ and $i_{\mathrm{pl}} = 10$, respectively. According to the eigenmode decomposition in Fig. S4A, the electric fields at $i_{\mathrm{pl}} = 1$ and $i_{\mathrm{pl}} = 5$ as well as $i_{\mathrm{pl}} = 12$ and $i_{\mathrm{pl}} = 8$ exhibit the same sign, which should result in a constructive interference of the incoming laser field and the near field of the mid-gap mode at $i_{\mathrm{pl}} = 5$ and $i_{\mathrm{pl}} = 8$. Indeed, looking at the FDTD response in Fig. S5A at an excitation energy of ~1.8 eV, there is significant intensity at $i_{\mathrm{pl}} = 5$

and $i_{\mathrm{pl}} = 8$ that contributes to photoemission, as shown by the experimentally measured data as well as by the yield simulations (Fig. 4a, Main Manuscript).

For the sake of completeness, we also show the effect of selective excitation of the outermost nanoslit resonator $i_{\mathrm{pl}} = 1$. As can be seen in Fig. S5B, the intensity decreases from nanoslit resonator $i_{\mathrm{pl}} = 1$ via $i_{\mathrm{pl}} = 3$ to $i_{\mathrm{pl}} = 5$ and then further into the bulk, while the nanoslit resonators $i_{\mathrm{pl}} = 2$ and $i_{\mathrm{pl}} = 4$ in between show an abrupt drop in intensity. This resembles the behavior of mid-gap modes. However, a selective excitation of a single resonator does not allow to discriminate between the excitation of the even- and odd-parity mid-gap mode. Consequently, both are excited simultaneously. Since they add up constructively on the left side of the chain there is, due to the opposite parity, hardly any intensity on the right side of the SSH chain. Although they are expected to be spectrally separated by a few tens of meV, they still exhibit sufficient spectral overlap to cancel out themselves on the right side of the chain. The superposition of both mid-gap modes also explains why the spectral intensity at $i_{\mathrm{pl}} = 1$ is shifted to lower energies compared to the spectral intensity at the same resonator under wide-field excitation conditions. The reason is that the energetically lower lying odd-parity mid-gap mode pulls the spectral weight to lower energies when both mid-gap modes are excited simultaneously. In summary, it can be concluded that the dip in the measured and simulated photoemission yield at nanoslit resonators $i_{\mathrm{pl}} = 3$ and $i_{\mathrm{pl}} = 10$ is due to the interference of incident laser light and the even-parity mid-gap mode near-field distribution as determined by the Zak phase.

## Eigenstates of the excitonic SSH chain in NTT and TT configuration

Here, we show in Fig. S6 and Fig. S7 the real part of the site-resolved wave functions of the excitonic SSH chains in nontrivial (NTT, left) and trivial (TT, right) configuration, divided into the six energetically lower and the six energetically higher states, respectively. The wave functions were retrieved by diagonalizing equation (1) in the Main Manuscript. In this representation, the alternating parity from eigenstate to eigenstate is clearly visible. We point out once again that due to the short chain length of twelve two-level systems, there is a wave function overlap of edge states which are mainly localized at the left and the right chain ends.

Hybridization of these edge states results in the odd- and even-parity mid-gap states at $E = 1.840$ eV and $E = 1.878$ eV, respectively, in the NTT configuration.

## Eigenmodes of the plasmonic SSH chain in NTT configuration

For the sake of completeness, we show all mode patterns (intensity) of the COMSOL eigenmode decomposition of the plasmonic SSH chain in NTT configuration in Fig. S8.

## Impact of chain cross talk in the PEEM experiment

In the main manuscript, it was found that the photoemission hot spots of an NTT chain at nanoslit resonators $i_{\mathrm{pl}} = 5$ and $i_{\mathrm{pl}} = 8$ are brighter than in simulated photoemission patterns based on the FDTD response function of a single NTT chain (Fig. 4A). More precisely, in the case of the PEEM experiment, these hot spots are brighter than the hot spots at the outer nanoslit resonators $i_{\mathrm{pl}} = 1$ and $i_{\mathrm{pl}} = 12$, which seems strange according to the expected field concentration of the edge-state mode at the chain ends. In the case of simulated photoemission on a single NTT chain, the outer nanoslit resonators $i_{\mathrm{pl}} = 1$ and $i_{\mathrm{pl}} = 12$ should exhibit the highest photoemission yield when the edge-state mode is excited from the far field.

The deviation from the expected to the actual measured photoemission hot spot pattern can be explained by cross talk between different SSH chains. For the PEEM experiment, SSH chains with varying parameters were fabricated in close proximity on the Au microplatelet so that several chains can be measured in the same field of view of the photoemission electron microscope. The chains were typically fabricated in parallel alignment (Fig. S9), as in an optical grating, with an approximate interchain distance around $D = 1.5$ and $D = 1.6$ µm. Under laser excitation, these chains also function as an injection source for surface-plasmon polaritons (SPPs) propagating on the surface of the Au microplatelet. The electromagnetic field of an SPP can then interfere with the field of a resonant chain mode, so that the local field responsible for the photoemission deviates from the local field of a resonant chain mode.

To demonstrate the influence of this so-called cross talk on the response function of an SSH chain, we show in Fig. S9 the FDTD response function of a single SSH chain in nontrivial (NTT) topology (see also Fig. S5A) in comparison to the FDTD response function of an SSH chain in nontrivial topology, when additionally an SSH chain in trivial (TT) topology and homogeneous (HT) topology are in close proximity. The inter-chain distance *D* increases from *D* = 1.3 µm (Fig. S9B) to *D* = 1.5 µm (Fig. S9C) and finally to *D* = 1.7 µm (Fig. S9D). Even if a mode hot spot is recognizable at the outer nanoslit resonators $i_{\mathrm{pl}} = 1$ and $i_{\mathrm{pl}} = 12$ of the NTT chain around an energy of *E* = 1.8 eV despite the presence of other chains (Fig. S9B–D), which indicates the edge-state mode, the exact spectral position and in particular the remaining behavior of the spatially-resolved response function (here in the form of the intensity $|E|^2$) differs considerably from the response function of the individual, isolated NTT chain (Fig. S9A). In particular, the case of *D* = 1.7 µm (Fig. S9D) shows for an energy of around 1.9 eV, just within the laser spectrum, that the local field intensity of the NTT chain at the inner nanoslit resonators $i_{\mathrm{pl}} = 5$ and $i_{\mathrm{pl}} = 8$ is higher than at the outer nanoslit resonators $i_{\mathrm{pl}} = 1$ and $i_{\mathrm{pl}} = 12$. Considering that the photoelectron yield depends nonlinearly on the local field intensity, the observed deviation of the measured photoemission hot spot pattern from the simulated pattern in the center of the NTT chain (Fig. 4A) can in principle be explained by the interference of the local edge-state mode with a propagating SPP field induced by neighboring chains.

## Determining the nonlinear order in photoemission

The dependence of the photoemission process on electric field polarization and its nonlinearity were investigated by measuring the PEEM yield as a function of the electric field polarization orientation. Figure S10 shows the spatially integrated, background-subtracted, and normalized PEEM yield of a nanoslit SSH chain with a nontrivial (A) and a trivial configuration (B). The angle $\alpha$ defines the relative orientation between the linear polarization of the excitation pulse(s) and the nanoslit chain. For $\alpha = 0°$ and $\alpha = 180°$ the polarization is aligned parallel to the long axis of the nanoslits and the chain, whereas at $\alpha = 90°$ (dashed line) the polarization is oriented parallel to the short axis of each nanoslit. The spatially integrated PEEM yield of both chains reaches a maximum at $\alpha = 90°$, consistent with the required electric field polarization along the short axis of the nanoslits for resonant excitation.

Due to the photon energy of $E_L = 1.837$ eV (675 nm), photoemitted electrons can only be generated via a nonlinear photoemission process. The order of the photoemission process can be determined from the polarization-dependent PEEM yield by fitting a modified version of Malus´ law,

$$y(\alpha) = a + b(\cos(\alpha + c))^{2N}, \quad \text{(S1)}$$

where *N* accounts for the nonlinearity of the photoemission process, *a* is a residual signal offset, *b* a yield scaling factor for the cosine function, and *c* is an offset angle for the orientation angle $\alpha$. The fit results (solid lines in Fig. S10) yield $N = 3.2$ for both configurations, indicating that, on average, three absorbed photons are required for photoemission.

## Aspects of nanoslit coupling

Impact of the slit width on resonator losses and coupling strength

In Fig. S11A, we show the scattering signal of nanoslit monomers with a slit width of 50 nm (black curve), 20 nm (red curve), and 12 nm (blue curve) as retrieved from FDTD simulations. For the resonator with the smallest slit width, we had to reduce the slit length *L* from *L* = 100 nm to *L* = 80 nm to keep the resonance energy within the spectral region of interest.

The nanoslit resonator with parameters *L* = 100 nm and w = 50 nm (black curve) is the one presented as a building block for the SSH chains in the Main Manuscript. Reducing the slit width to *w* = 20 nm introduces a small red shift of the resonance energy of about 100 meV. The reduction of the slit width by more than a factor of two almost halves the linewidth (full width at half maximum, FWHM) of the resonance, from 396 meV to 232 meV, which corresponds to an increase of the nanoslit's quality factor from *Q* = 4.7 to *Q* = 7.5. The fact that the reduction in optical losses is due to the reduced width of the nanoslit resonator, and not due to the red shift, is demonstrated by a further reduction in width to *w* = 12 nm, where the also shorter length of *L* = 80 nm of the resonator prevents the resonance energy from shifting further to the red: As a result, the FWHM is reduced to 169 meV and the quality factor therefore continues to increase to *Q* = 10.6.

In Fig. S11B, we show the coupling strengths of the corresponding nanoslit dimers as a function of the bridge size *b*. The results were retrieved via FDTD simulations in the same way as described in the Main Manuscript in the caption of Fig. 2. The highest coupling strength is achieved by the nanoslit dimers with the broadest slit width of $w$ = 50 nm. With a bridge size of $b$ = 12 nm, as used for the simulations and experiments in the Main Manuscript, the coupling strength gradually decreases as the nanoslit width decreases: from 238 meV to 183 meV for $w$ = 20 nm, and finally to 139 meV for the smallest width of $w$ = 12 nm. The coupling strength between two closely spaced nanoslit resonators appears to diminish as the length of their opposing facets is reduced, consistent with a reduced spatial overlap between the charge currents and densities of the monomeric entities.

We note that a nanoslit width of $w$ = 20 nm combined with a dimer bridge size of $b$ = 12 nm still leads to a coupling strength that is 10% of the resonance energy. Nanoslit resonators with a slit width of $w$ = 12 nm would then no longer be in the ultra-strong coupling regime with the bridge sizes of $b$ = 12 nm and $b$ = 24 nm used in the main manuscript. If the reduced FWHM of the 12 nm wide nanoslit resonators is included in the discussion, the peak splitting $2\hbar g$ = 278 meV in the case of these dimers is greater than the FWHM of the individual monomer entities of 169 meV. According to the conservative rule of thumb that the peak splitting should be larger than the line width of the individual monomers, a nanoslit width of $w$ = 12 nm allows to enter the strong coupling regime for a bridge size of $b$ = 12 nm.

To conclude, the width of the nanoslit resonators is, in addition to the dimer's bridge size, an essential parameter for tuning both the coupling strength and line width of the resonators.

## Assessment of coupling strength

According to Kockum and co-workers (*24*), the ultra-strong coupling (USC) regime is, in contrast to the strong coupling regime, defined by the ratio of the coupling strength to the bare excitation energies rather than by the relation to system losses. The question with this interaction regime is whether certain terms of the interaction term of the Hamilton operator can be neglected or not. The answer to this question depends only on the coupling strength, but not on the losses of the system. A system can therefore enter the USC regime even if strong coupling is not spectroscopically observable due to large losses. The relevant figure of merit is $\eta$, i.e., the above-mentioned ratio of the coupling strength to the resonance energy of the system's constituents. In

our case, we find that the rule-of-thumb criterion $\eta > 0.1$ is formally fulfilled for dimers of nanoslit resonators ($w = 50$ nm) up to a bridge size of $b \approx 20$ nm (see Fig. 2A, Main Manuscript).

However, the labeling of such regimes is commonly done for light–matter interactions which are described by the quantum Rabi model. Nevertheless, we label our coupling regime as ultra-strong because Kockum and co-workers also describe the application of the quantum Rabi model to systems of two coupled harmonic oscillators (*24*). In previous experimental work, some of us were able to exploit the fact that localized surface plasmons of nanoslits can be described as quantum mechanical harmonic oscillators (*37*). Against this background, we label the coupling strength in the Main Manuscript as *ultra-strong*.

## Extension of the plasmonic SSH chain into two spatial dimensions

As stated in the Main Manuscript, the nanoslit design of the monomeric entities of the SSH chain offers the possibility of extending the system to two spatial dimensions. And this in turn offers the possibility of obtaining higher-order topological states. Therefore, we have conducted FDTD simulations of the coupling strength for the two spatial configurations of nanoslit resonators, i.e., H-type and J-type dimers, that would occur in a 2D array. The H-type dimer forms the basis for our work on 1D SSH chains in the Main Manuscript and it exhibits electric dipole moments that couple head-to-head (inset Fig. 2A, Main Manuscript). According to the terminology that has been established with molecular excitons, the J-type dimer exhibits head-to-tail coupling of the electric dipole moments, i.e., the long sides of the resonators face each other.

In Fig. S12, we show the coupling strength of an H-type dimer and a J-type dimer as a function of the bridge size $b$. Coupling strengths were retrieved in the same way as already discussed for Figure 2A in the Main Manuscript and Figure S11 in the Supplementary Text. Here, the nanoslit resonators exhibit a length $L = 80$ nm and a width $w = 12$ nm. Using these parameters, the J-type dimer offers a larger coupling strength than the H-type dimer. We also note that the coupling strength decreases more rapidly with bridge size for the J-type dimer compared to the H-type dimer.

The results of Fig. S12 form the basis for building a 2D SSH array of nanoslit resonators. To ensure that the same alternation of coupling strength occurs in the spatial direction of J-type

coupling and H-type coupling, it is required to adjust the bridge size $b$ accordingly. As an example, choosing bridge sizes of $b_1$ = 36 nm and $b_2$ = 12 nm in H direction requires bridge sizes of $b_1$ = 39 and $b_2$ = 19 nm in J direction. These parameters give a ratio of coupling strengths of approximately $v(b_1)/w(b_2)$ = 69 meV/139 meV = 0.5, which reflects a trade-off between edge state localization and avoidance of the dimeric limit. Note that the quantity $w$ represents the coupling strength of the tight-binding model in this equation, and not the nanoslit resonator width.

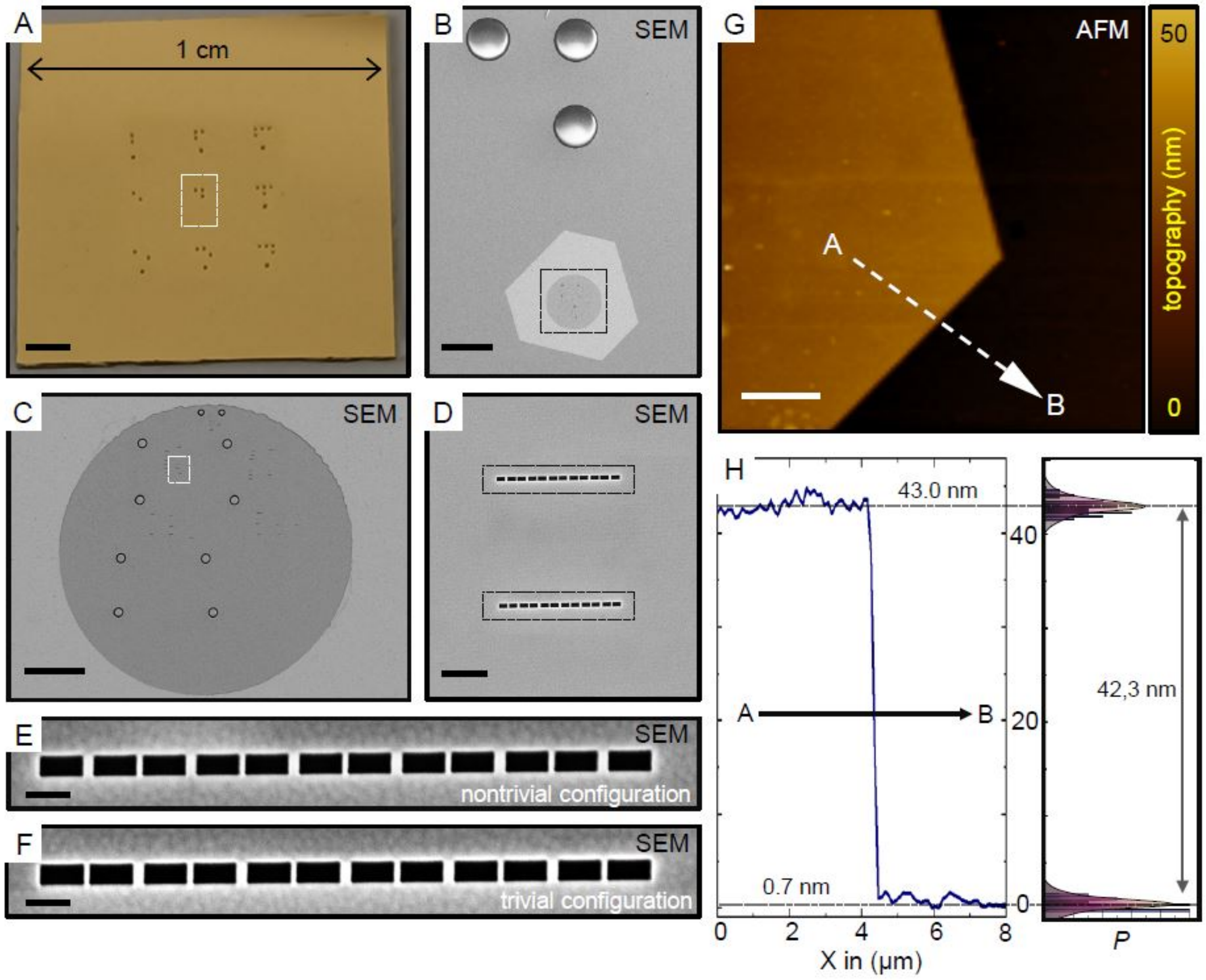


**Fig. S1.**

**Sample layout and gold microplatelet thickness**. (**A**) Camera image of the sample used for wide-field PEEM experiments. Since a mask was used before the evaporation process, a specific 3×3 hole pattern matrix was created on the sample, providing circular holes with diameters of up to 100 µm for the microplatelet transfer. A monocrystalline gold microplatelet was placed on such a hole to provide a monocrystalline gold layer on top of the pure glass substrate for optical measurements. The scale bar is 1 mm. (**B**) SEM scan of one matrix spot on the sample, (marked in (**A**) showing a positioned Au microplatelet on a prepared hole. The scale bar is 100 µm. (**C**) Zoom-in SEM scan of the marker area in (**B**). Marker structures (ring-like objects) were cut into the 2D monocrystalline gold using Ga FIB milling. These structures, measuring up to 5 µm, were used to locate the position of the SSH chains in the PEEM. The scale bar is 20 µm. (**D**) SEM image of a nontrivial and trivial SSH chain, as a zoom-in of the marked position in **c** next to a marker. The scale bar is 500 nm. (**E**) and (**F**) Close-up SEM scans of the nontrivial and trivial SSH chain configurations, respectively. The scale bars are each 100 nm. (**G**) AFM scan of a corner of the used monocrystalline Au microplatelet used in (**B-F**). The scale bar is 1 µm. (**H**) Analysis of the height profile from the line cut shown in (**G**). The thickness of the microplatelet was estimated from the histogram to be 42.3 nm.

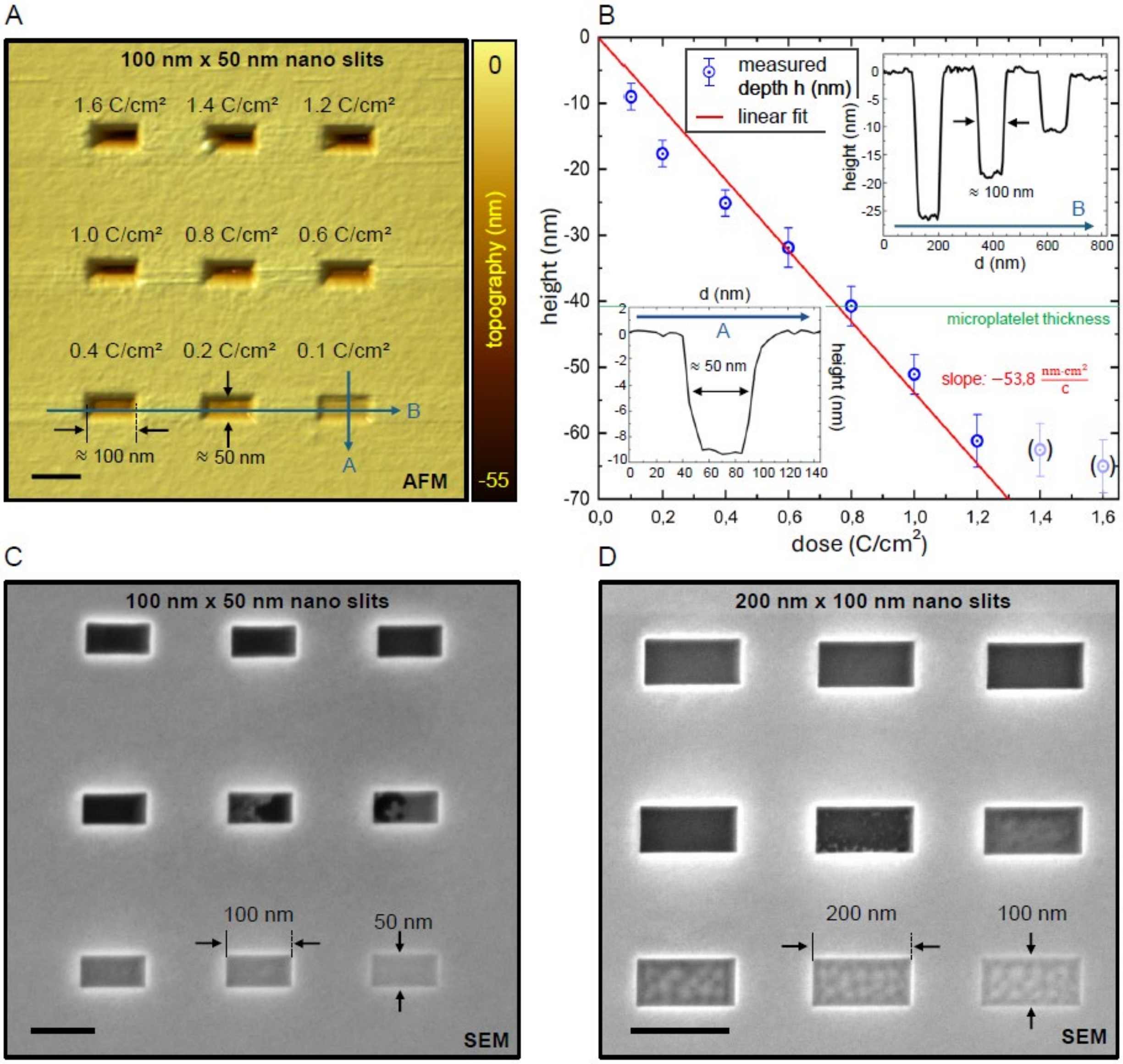


**Fig. S2.**

**Nanoslit fabrication and dose tests.** (**A**) 3D AFM scan of nine helium-ion-milled rectangular nanoslits, each measuring 50 nm in width and 100 nm in length. To check the depth evolution of the fabricated nanoslits inside the Au microplatelet with increasing He ion doses, nine nanoslits have been milled out using doses ranging from 0.1 C/cm² to 1.6 C/cm². (**B**) Measured nanoslit depths of the dose test structures from (**A**). Increasing He ion doses lead to larger depths of the created nanoslit. As displayed in (**B**), a linear behavior can be found, exhibiting a slope of the depth-dose relation of approximately -53.8 $(\mathbf{nm} \cdot \mathbf{cm^2})/\mathbf{C}$. A saturation behavior above 1.2 C/cm² (excluded data) occurs due to a limited resolution of the AFM with respect to the AFM tip radius. (**C**) SEM image of the nine nanoslits presented in (**A**). As visible from the contrasts, the dose-depth relation found in (**A**), (**B**) could also be found in the SEM image. (**D**) SEM scan of nanoslits twice as large compared to those in (**A**-**C**), providing the same aspect ratio of 2:1.

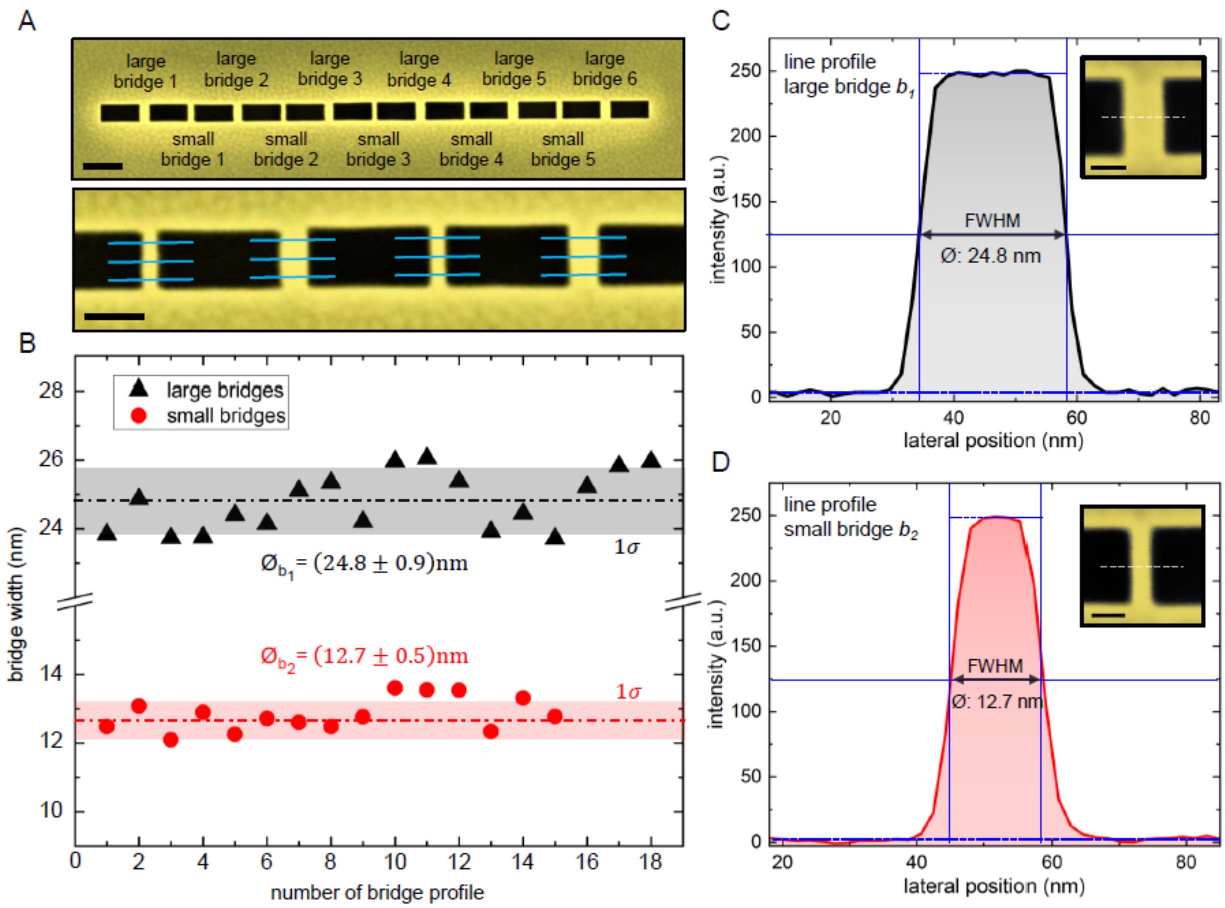


**Fig. S3.**

**Evaluation of bridge widths of an SSH chain**. (**A**) SEM scan of a complete NTT SSH chain with labeled bridges (top panel). To evaluate the large and small bridge widths of $b_1$ and $b_2$, respectively, three contrast line cuts have been extracted from a zoom-in SEM image (bottom panel) for each bridge to obtain average values. (**B**) Measured width values for the smaller (red dots) and larger bridges (black triangles). Both the large and small bridge widths exhibit a narrow distribution around their mean values of $(24.8 \pm 0.9)$ nm and $(12.7 \pm 0.5)$ nm, respectively. Additionally, the $1\sigma$ interval is displayed for both measurement series. (**C, D**) Exemplary line cuts for the analysis of the bridge widths for the large bridges $b_1$ (**C**) and for the small bridges $b_2$ (**D**). The values of the bridge widths were determined by the full width at half maximum (FWHM) of each of the line cuts. The insets in (**C**) and (**D**) show close-up SEM scans of the corresponding bridge $b_1$ and $b_2$.

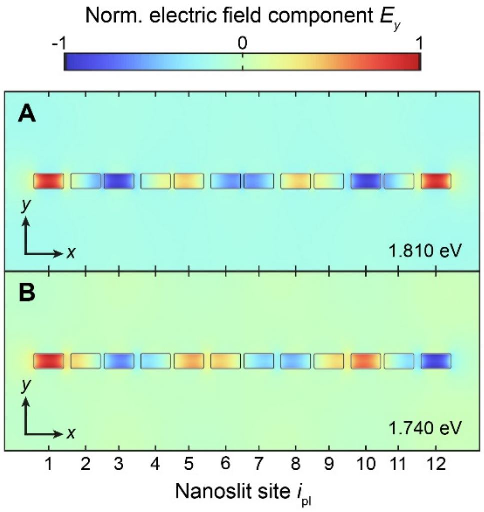


**Fig. S4.**

**Real-valued $E_y$ component of the two plasmonic mid-gap modes retrieved from COMSOL simulations**. (**A**) Even-parity mid-gap mode. (**B**) Odd-parity mid-gap mode. The field strength is normalized with respect to each specific eigenmode, and the near-field distributions are retrieved at half of the nanoslit height.

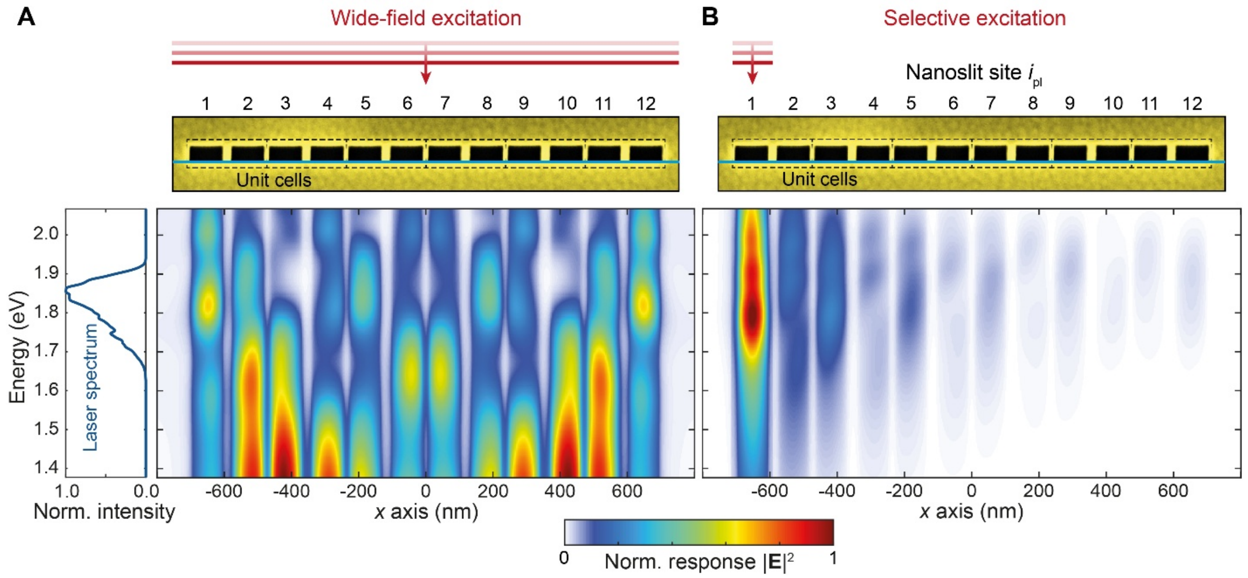


**Fig. S5.**

**Spatially resolved absolute square of the FDTD response function for different excitation conditions**. (**A**) Wide-field excitation with a plane-wave source, i.e., a total-field scattered-field (TFSF) source in Lumerical FDTD, spanning the entire chain. Additionally, the experimental laser spectrum is shown on the left. (**B**) Selective excitation of the outermost nanoslit resonator on the left side by spatially restricting the TFSF plane-wave source to that specific resonator. Data is collected along the light-blue line, 3 nm above the gold surface. All $|E|^2$ data is normalized to the maximum of the wide-field excitation response.

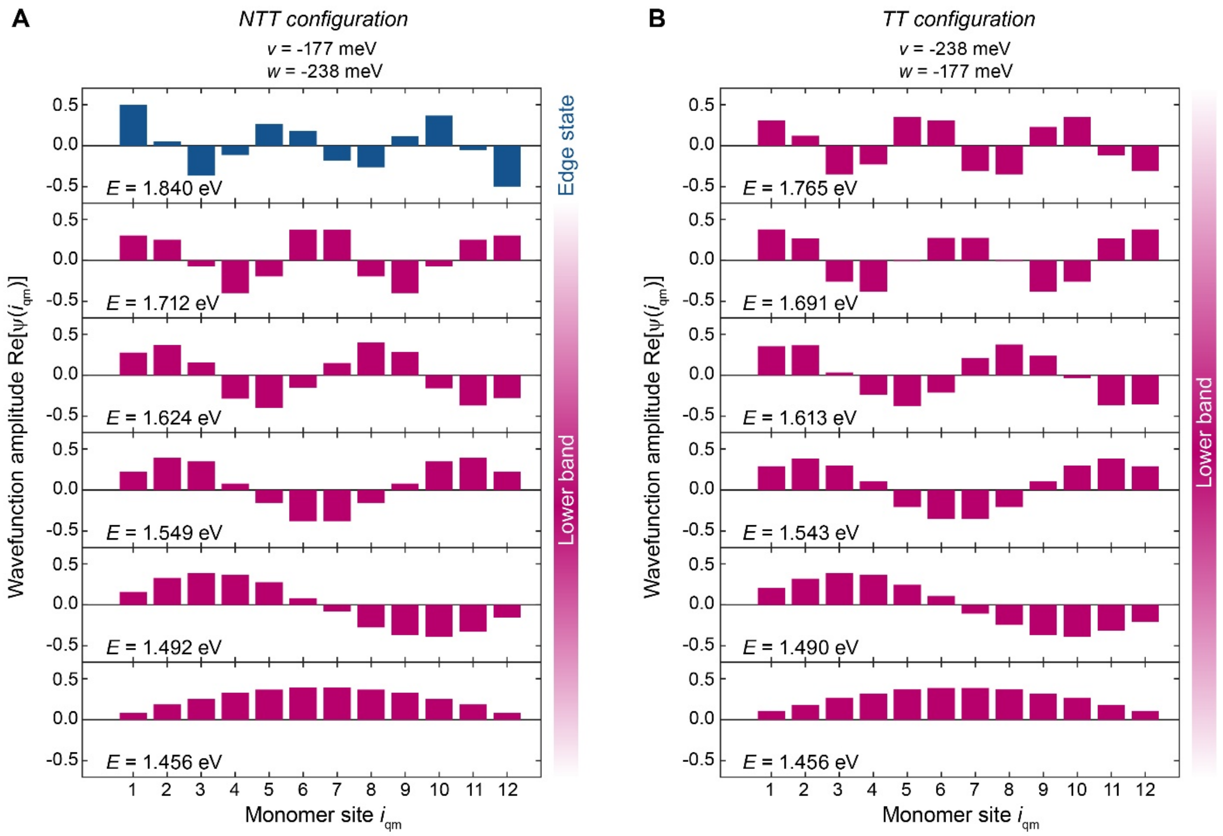


**Fig. S6.**

**Site-resolved lower-band and edge-state wave functions (real part) of the excitonic SSH chain**. (**A**) Nontrivial (NTT) chain configuration. (**B**) Trivial (TT) chain configuration. Derived by diagonalization of equation (1) in the main manuscript.

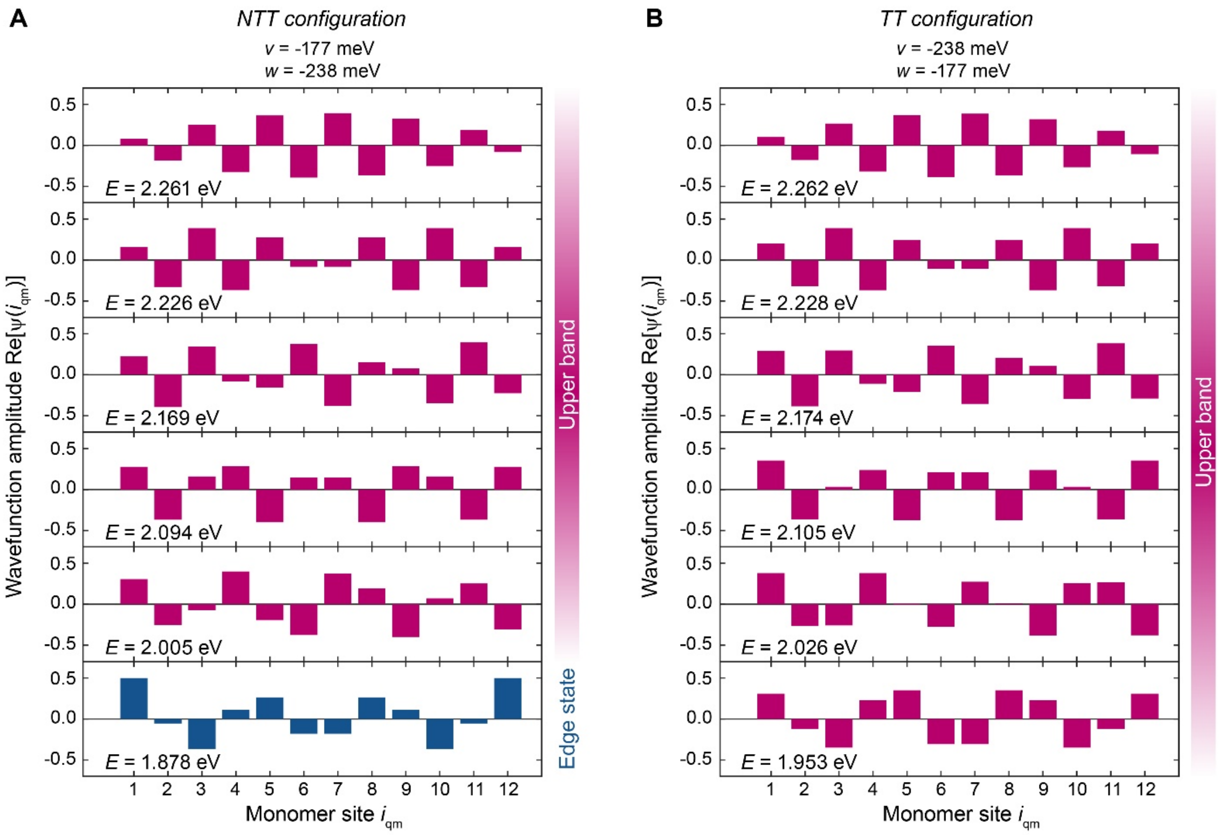


**Fig. S7.**

**Site-resolved edge-state and upper-band wave functions (real part) of the excitonic SSH chain**. (**A**) Nontrivial (NTT) chain configuration. (**B**) Trivial (TT) chain configuration. Derived by diagonalization of equation (1) in the main manuscript.

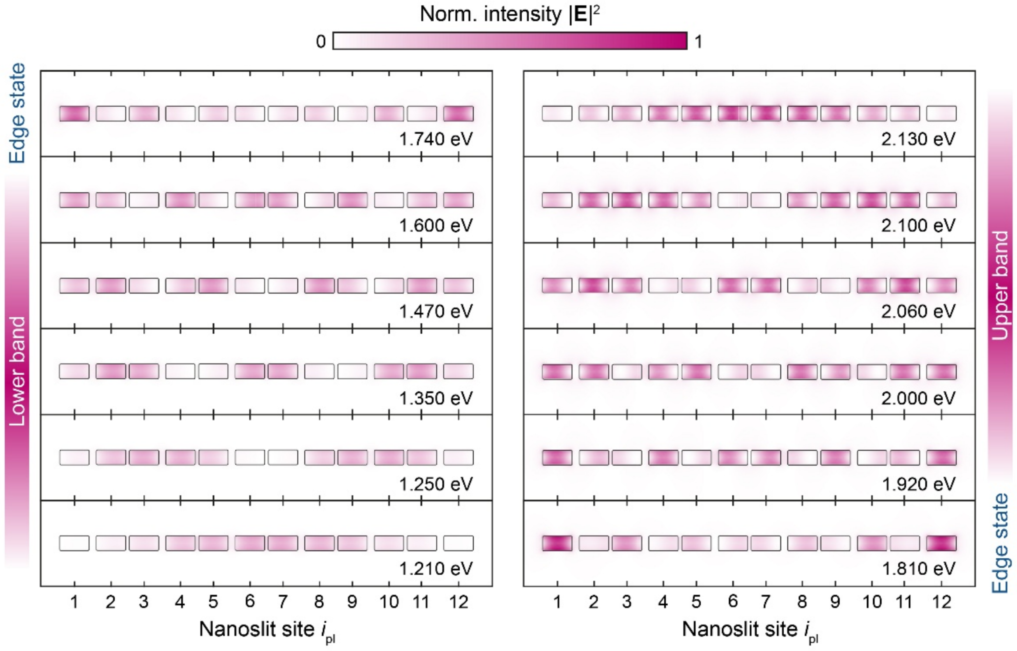


**Fig. S8.**

**Intensity distribution of the plasmonic SSH chain eigenmodes for the nontrivial (NTT) configuration retrieved from a COMSOL eigenmode decomposition**. All mode patterns are normalized to a global maximum. Data is retrieved from a lateral monitor located at half the height of the nanoslits. The bridge sizes are $b_1$ = 24 nm and $b_2$ = 12 nm.

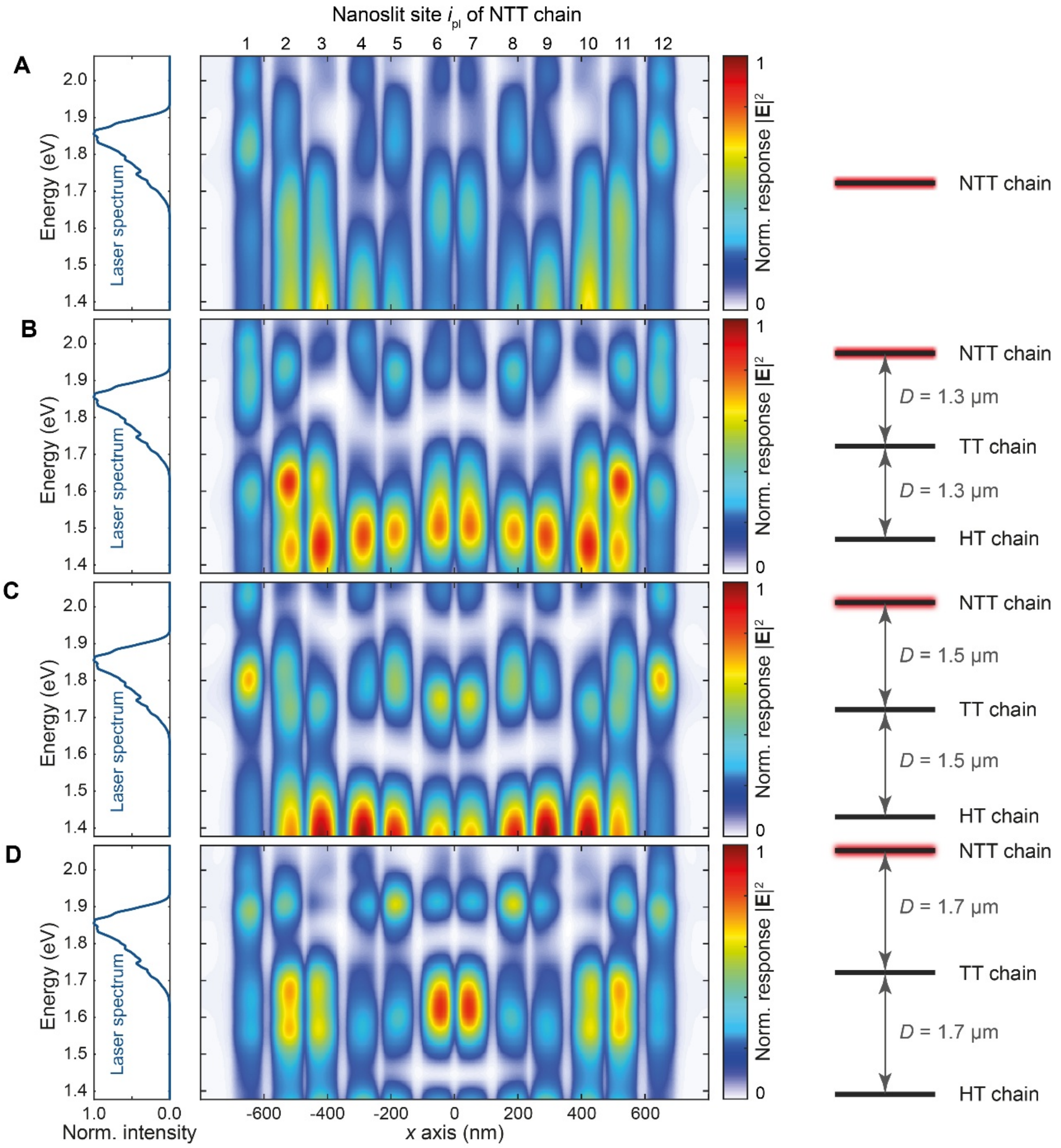


**Fig. S9.**

**Impact of chain cross talk on the NTT chain response function.** Wide-field excitation FDTD response function (intensity $|E|^2$) of the NTT chain ($b_1$ = 24 nm and $b_2$ = 12 nm) for four different scenarios: (**A**) A single NTT, a NTT chain with a nearby TT chain ($b_1$ = 12 nm and $b_2$ = 24 nm) and HT chain ($b_1 = b_2$ = 16 nm) where the interchain distance amounts to (**B**) $D$ = 1.3 µm, (**C**) $D$ = 1.5 µm, and (**D**) $D$ = 1.7 µm. Data is collected along the light-blue line shown in Fig. S5, 3 nm above the gold surface. All $|E|^2$ data is normalized to the case of $D$ = 1.5 µm.

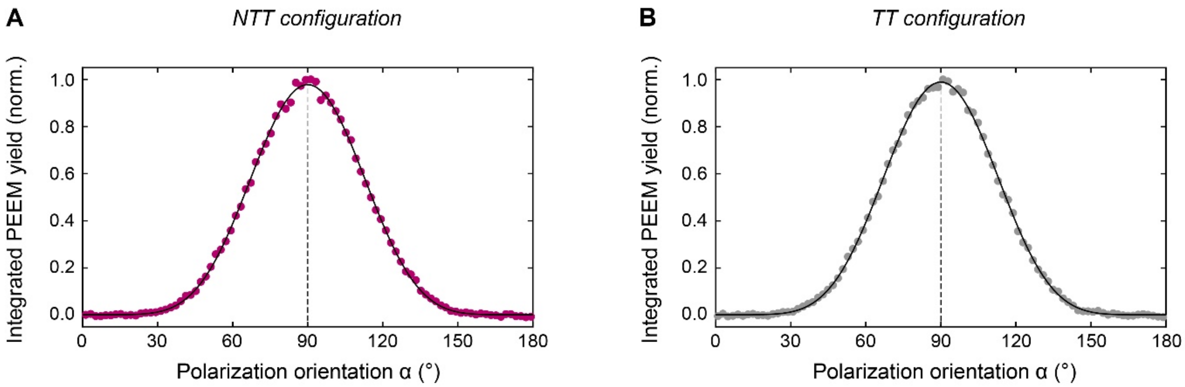


**Fig. S10.**

**PEEM yield polarization orientation dependency.** (**A**) Spatially integrated, background-subtracted, and normalized PEEM yield of the nontrivial configuration nanoslit chain (NTT, $v = -177$ meV and $w = -238$ meV) dependent on the polarization angle of the excitation light field with respect to the nanoslit chain orientation. Fit according to equation S1 (solid line) reveals an $N = 3$ order of the photoemission process. The dashed line indicates the PEEM yield maximum at $\alpha = 90°$, i.e., for a polarization along the short axis of nanoslit. (**B**) Equivalent representation as in (a) for the trivial configuration (TT, $v = -238$ meV and $w = -177$ meV).

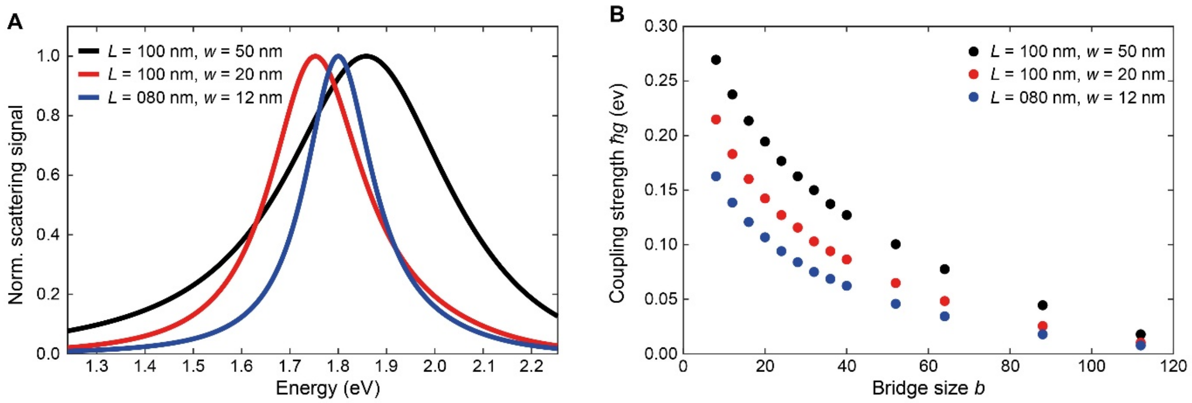


**Fig. S11.**

**Impact of the resonator width on optical losses and coupling strength.** (**A**) Nanoslit monomer scattering signals retrieved from FDTD simulations. All curves are normalized to their respective maximum. (**B**) Coupling strength of nanoslit dimers as a function of the bridge size *b* between the nanoslit resonators for different resonator length *L* and width *w*. The coupling strength is determined in the same way as described in Fig. 2 of the Main Manuscript.

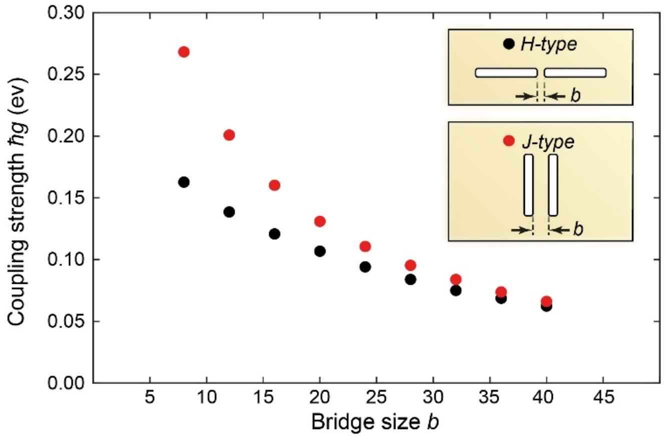


**Fig. S12.**

**Prerequisites for extending the nanoslit SSH chain to two spatial dimensions.** Coupling strength of H-type and J-type nanoslit dimers as a function of the bridge size *b* between the nanoslit resonators. In this case, the nanoslit resonator length is $L$ = 80 nm and the width $w$ = 12 nm. The coupling strength is determined in the same way as described in Fig. 2 of the Main Manuscript and Fig. S11B in the Supplementary Text.

## REFERENCES AND NOTES


1. M. S. Rider, S. J. Palmer, S. R. Pocock, X. Xiao, P. Arroyo Huidobro, V. Giannini, A perspective on topological nanophotonics: Current status and future challenges. *J. Appl. Phys.* **125**, 120901 (2019).

2. J. K. Asbóth, L. Oroszlány, A. Pályi, *A Short Course on Topological Insulators*, vol. 919 of *Lecture Notes in Physics* (Springer International Publishing, 2016); http://link.springer.com/10.1007/978-3-319-25607-8.

3. W. P. Su, J. R. Schrieffer, A. J. Heeger, Solitons in polyacetylene. *Phys. Rev. Lett.* **42**, 1698–1701 (1979).

4. Z. Fedorova, C. Jörg, C. Dauer, F. Letscher, M. Fleischhauer, S. Eggert, S. Linden, G. von Freymann, Limits of topological protection under local periodic driving. *Light Sci Appl* **8**, 63 (2019).

5. L. J. Maczewsky, M. Heinrich, M. Kremer, S. K. Ivanov, M. Ehrhardt, F. Martinez, Y. V. Kartashov, V. V. Konotop, L. Torner, D. Bauer, A. Szameit, Nonlinearity-induced photonic topological insulator. *Science* **370**, 701–704 (2020).

6. M. C. Rechtsman, J. M. Zeuner, Y. Plotnik, Y. Lumer, D. Podolsky, F. Dreisow, S. Nolte, M. Segev, A. Szameit, Photonic Floquet topological insulators. *Nature* **496**, 196–200 (2013).

7. H. Groß, J. M. Hamm, T. Tufarelli, O. Hess, B. Hecht, Near-field strong coupling of single quantum dots. *Sci. Adv.* **4**, eaar4906 (2018).

8. R. Chikkaraddy, B. de Nijs, F. Benz, S. J. Barrow, O. A. Scherman, E. Rosta, A. Demetriadou, P. Fox, O. Hess, J. J. Baumberg, Single-molecule strong coupling at room temperature in plasmonic nanocavities. *Nature* **535**, 127–130 (2016).

9. M. Hensen, T. Heilpern, S. K. Gray, W. Pfeiffer, Strong coupling and entanglement of quantum emitters embedded in a nanoantenna-enhanced plasmonic cavity. *ACS Photonics* **5**, 240–248 (2018).

10. Q. Yan, E. Cao, Q. Sun, Y. Ao, X. Hu, X. Shi, Q. Gong, H. Misawa, Near-field imaging and time-domain dynamics of photonic topological edge states in plasmonic nanochains. *Nano Lett.* **21**, 9270–9278 (2021).

11. Q. Yan, B. Zhao, Q. Lyu, Y. Li, S. Chu, C. Lu, X. Hu, C. T. Chan, Q. Gong, Near-field imaging of synthetic dimensional integrated plasmonic topological Harper nanochains. *Nat. Commun.* **16**, 2592 (2025).

12. Y. Moritake, M. Ono, M. Notomi, Far-field optical imaging of topological edge states in zigzag plasmonic chains. *Nanophotonics* **11**, 2183–2189 (2022).

13. I. S. Sinev, I. S. Mukhin, A. P. Slobozhanyuk, A. N. Poddubny, A. E. Miroshnichenko, A. K. Samusev, Y. S. Kivshar, Mapping plasmonic topological states at the nanoscale. *Nanoscale* **7**, 11904–11908 (2015).

14. S. R. Pocock, X. Xiao, P. A. Huidobro, V. Giannini, Topological plasmonic chain with retardation and radiative effects. *ACS Photonics* **5**, 2271–2279 (2018).

15. L. Orsini, H. Herzig Sheinfux, Y. Li, S. Lee, G. M. Andolina, O. Scarlatella, M. Ceccanti, K. Soundarapandian, E. Janzen, J. H. Edgar, G. Shvets, F. H. L. Koppens, Deep subwavelength topological edge state in a hyperbolic medium. *Nat. Nanotechnol.* **19**, 1485–1490 (2024).

16. G. Veronis, S. Fan, Modes of subwavelength plasmonic slot waveguides. *J. Lightwave Technol.* **25**, 2511–2521 (2007).

17. E. Moreno, F. J. Garcia-Vidal, S. G. Rodrigo, L. Martin-Moreno, S. I. Bozhevolnyi, Channel plasmon-polaritons: Modal shape, dispersion, and losses. *Opt. Lett.* **31**, 3447–3449 (2006).

18. M. Proctor, M. Blanco de Paz, D. Bercioux, A. García-Etxarri, P. Arroyo Huidobro, Higher-order topology in plasmonic Kagome lattices. *Appl. Phys. Lett.* **118**, 091105 (2021).

19. Á. Buendía, J. A. Sánchez-Gil, V. Giannini, W. L. Barnes, M. S. Rider, Long-range molecular energy transfer mediated by strong coupling to plasmonic topological edge states. *Nanophotonics* **13**, 4555–4568 (2024).

20. D. Rossouw, G. A. Botton, Resonant optical excitations in complementary plasmonic nanostructures. *Opt. Express* **20**, 6968–6973 (2012).

21. D. Lee, D.-S. Kim, Light scattering of rectangular slot antennas: Parallel magnetic vector vs perpendicular electric vector. *Sci. Rep.* **6**, 18935 (2016).

22. P. Biagioni, J. S. Huang, B. Hecht, Nanoantennas for visible and infrared radiation. *Rep. Prog. Phys.* **75**, 024402 (2012).

23. H. Mizobata, K. Ueno, H. Misawa, H. Okamoto, K. Imura, Near-field spectroscopic properties of complementary gold nanostructures: Applicability of Babinet's principle in the optical region. *Opt. Express* **25**, 5279–5289 (2017).

24. A. Frisk Kockum, A. Miranowicz, S. De Liberato, S. Savasta, F. Nori, Ultrastrong coupling between light and matter. *Nat. Rev. Phys.* **1**, 19–40 (2019).

25. T. Zentgraf, T. P. Meyrath, A. Seidel, S. Kaiser, H. Giessen, C. Rockstuhl, F. Lederer, Babinet's principle for optical frequency metamaterials and nanoantennas. *Phys. Rev. B* **76**, 033407 (2007).

26. N. Liu, S. Kaiser, H. Giessen, Magnetoinductive and electroinductive coupling in plasmonic metamaterial molecules. *Adv. Mater.* **20**, 4521–4525 (2008).

27. M. Hentschel, T. Weiss, S. Bagheri, H. Giessen, Babinet to the half: Coupling of solid and inverse plasmonic structures. *Nano Lett.* **13**, 4428–4433 (2013).

28. A. V. Pisliakov, T. Mančal, G. R. Fleming, Two-dimensional optical three-pulse photon echo spectroscopy. II. Signatures of coherent electronic motion and exciton population transfer in dimer two-dimensional spectra. *J. Chem. Phys.* **124**, 234505 (2006).

29. B. Huber, S. Pres, E. Wittmann, L. Dietrich, J. Lüttig, D. Fersch, E. Krauss, D. Friedrich, J. Kern, V. Lisinetskii, M. Hensen, B. Hecht, R. Bratschitsch, E. Riedle, T. Brixner, Space- and time-resolved UV-to-NIR surface spectroscopy and 2D nanoscopy at 1 MHz repetition rate. *Rev. Sci. Instrum.* **90**, 113103 (2019).

30. M. Dąbrowski, Y. Dai, H. Petek, Ultrafast microscopy: Imaging light with photoelectrons on the nano–femto scale. *J. Phys. Chem. Lett.* **8**, 4446–4455 (2017).

31. T. J. Davis, D. Janoschka, P. Dreher, B. Frank, F.-J. Meyer Zu Heringdorf, H. Giessen, Ultrafast vector imaging of plasmonic skyrmion dynamics with deep subwavelength resolution. *Science* **368**, eaba6415 (2020).

32. J. Zak, Berry's phase for energy bands in solids. *Phys. Rev. Lett.* **62**, 2747–2750 (1989).

33. W. S. Gao, M. Xiao, C. T. Chan, W. Y. Tam, Determination of Zak phase by reflection phase in 1D photonic crystals. *Opt. Lett.* **40**, 5259–5262 (2015).

34. C. Liu, H. R. Wang, H. C. Ong, Determination of the Zak phase of one-dimensional diffractive systems with inversion symmetry via radiation in Fourier space. *Phys. Rev. B* **108**, 035403 (2023).

35. M. Xiao, Z. Q. Zhang, C. T. Chan, Surface impedance and bulk band geometric phases in one-dimensional systems. *Phys. Rev. X* **4**, 021017 (2014).

36. K. Chen, G. Razinskas, H. Vieker, H. Gross, X. Wu, A. Beyer, A. Gölzhäuser, B. Hecht, High-Q, low-mode-volume and multiresonant plasmonic nanoslit cavities fabricated by helium ion milling. *Nanoscale* **10**, 17148–17155 (2018).

37. M. Aeschlimann, T. Brixner, A. Fischer, C. Kramer, P. Melchior, W. Pfeiffer, C. Schneider, C. Strüber, P. Tuchscherer, D. V. Voronine, Coherent two-dimensional nanoscopy. *Science* **333**, 1723–1726 (2011).

38. S. Pres, B. Huber, M. Hensen, D. Fersch, E. Schatz, D. Friedrich, V. Lisinetskii, R. Pompe, B. Hecht, W. Pfeiffer, T. Brixner, Detection of a plasmon-polariton quantum wave packet. *Nat. Phys.* **19**, 656–662 (2023).

39. E. J. R. Vesseur, R. de Waele, M. Kuttge, A. Polman, Direct observation of plasmonic modes in Au nanowires using high-resolution cathodoluminescence spectroscopy. *Nano Lett.* **7**, 2843–2846 (2007).

40. H. Saito, D. Yoshimoto, Y. Moritake, T. Matsukata, N. Yamamoto, T. Sannomiya, Valley-polarized plasmonic edge mode visualized in the near-infrared spectral range. *Nano Lett.* **21**, 6556–6562 (2021).

41. E. D. Palik, *Handbook of Optical Constants of Solids* (Academic Press, 1997).

42. P. B. Johnson, R. W. Christy, Optical constants of the noble metals. *Phys. Rev. B* **6**, 4370–4379 (1972).

43. D. Podbiel, P. Kahl, A. Makris, B. Frank, S. Sindermann, T. J. Davis, H. Giessen, M. H. Hoegen, F.-J. Meyer zu Heringdorf, Imaging the nonlinear plasmoemission dynamics of electrons from strong plasmonic fields. *Nano Lett.* **17**, 6569–6574 (2017).

44. D. L. Lyutov, K. V. Genkov, A. D. Zyapkov, G. G. Tsutsumanova, A. N. Tzonev, L. G. Lyutov, S. C. Russev, Synthesis and structure of large single crystalline silver hexagonal microplates suitable for micromachining. *Mater. Chem. Phys.* **143**, 642–646 (2014).

45. C. Schörner, S. Adhikari, M. Lippitz, A single-crystalline silver plasmonic circuit for visible quantum emitters. *Nano Lett.* **19**, 3238–3243 (2019).

46. X. Wu, R. Kullock, E. Krauss, B. Hecht, Single-crystalline gold microplates grown on substrates by solution-phase synthesis. *Cryst. Res. Technol.* **50**, 595–602 (2015).

47. J. Notte, B. Ward, N. Economou, An introduction to the helium ion microscope. *Micros. Today* **14**, 24–31 (2006).
